# Synthesis of ^19^F MRI Nanotracers by Dispersion Polymerization-Induced Self-Assembly of *N*-(2,2,2-Trifluoroethyl)acrylamide in Water

**DOI:** 10.1021/acs.biomac.2c00981

**Published:** 2022-10-17

**Authors:** Vyshakh M. Panakkal, Dominik Havlicek, Ewa Pavlova, Marcela Filipová, Semira Bener, Daniel Jirak, Ondrej Sedlacek

**Affiliations:** † Department of Physical and Macromolecular Chemistry, Faculty of Science, Charles University, Prague 2 128 40, Czech Republic; ‡ Department of Diagnostic and Interventional Radiology, Institute for Clinical and Experimental Medicine, Prague 140 21, Czech Republic; § Faculty of Health Studies, Technical University of Liberec, Studentská 1402/2, Liberec 461 17, Czech Republic; ∥ Institute of Macromolecular Chemistry, AS CR, Prague 6 162 06, Czech Republic

## Abstract

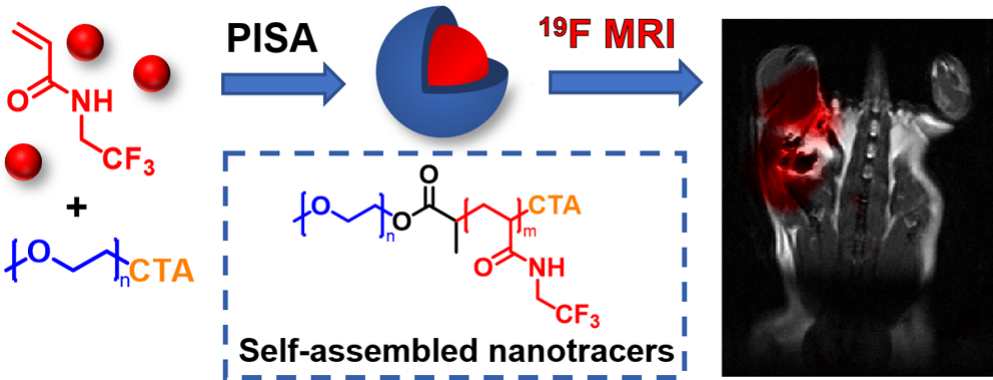

^19^F magnetic resonance imaging (MRI) using fluoropolymer tracers has recently emerged as a promising, non-invasive diagnostic tool in modern medicine. However, despite its potential, ^19^F MRI remains overlooked and underused due to the limited availability or unfavorable properties of fluorinated tracers. Herein, we report a straightforward synthetic route to highly fluorinated ^19^F MRI nanotracers *via* aqueous dispersion polymerization-induced self-assembly of a water-soluble fluorinated monomer. A polyethylene glycol-based macromolecular chain-transfer agent was extended by RAFT-mediated *N*-(2,2,2-trifluoroethyl)acrylamide (TFEAM) polymerization in water, providing fluorine-rich self-assembled nanoparticles in a single step. The resulting nanoparticles had different morphologies and sizes ranging from 60 to 220 nm. After optimizing their structure to maximize the magnetic relaxation of the fluorinated core, we obtained a strong ^19^F NMR/MRI signal in an aqueous environment. Their non-toxicity was confirmed on primary human dermal fibroblasts. Moreover, we visualized the nanoparticles by ^19^F MRI, both *in vitro* (in aqueous phantoms) and *in vivo* (after subcutaneous injection in mice), thus confirming their biomedical potential.

## 1 Introduction

Fluoropolymers stand out as an important class of high-performance materials with numerous commercial applications, ranging from construction materials through antiadhesive coatings and electronics to biomaterials.^1,2^ Fluorinated polymers have recently shown significant potential in medicine as metal-free diagnostic tracers for ^19^F magnetic resonance (MR) imaging (MRI).^3,4^ Although currently used clinical MRI techniques highlight the biodistribution of hydrogen nuclei (mainly from water and lipids), they suffer from a high background of omnipresent water. Conversely, ^19^F MRI accurately visualizes magnetically active natural fluorine atoms because there is virtually no fluorine background in the body, which enables a straightforward “hotspot” visualization of fluorinated tracers in an organism for diagnostic purposes. Furthermore, given the resonance frequency of ^19^F, which is very close to that of hydrogen, fluorinated tracers can be visualized on commercial MRI scanners with only minor radiofrequency coil adjustments.^5^ Accordingly, spectrally resolved MR can simultaneously provide both spatial and spectroscopic data and thus more complex information and be used with various systems for its advantages over conventional imaging, including higher sensitivity.^6,7^

Notwithstanding its potential, ^19^F MRI is still only relatively sparsely used, mostly due to the limited availability or unfavorable properties of fluorinated tracers. On the one hand, perfluorocarbons (PFCs) and perfluoro-crown ethers (PFCEs) benefit from their high fluorine content but exhibit an extremely hydrophobic character,^8,9^ thus requiring stabilization in nanoemulsions with surfactants. As a result, they display relative instability, limited biocompatibility, and suboptimal magnetic relaxations of fluorine atoms. On the other hand, fluorinated polymer materials open up countless possibilities regarding their macromolecular architecture and fluorine modifications.^3,5^ In particular, water-soluble semifluorinated polymers, such as poly(*N*-(2-((2,2,2-trifluoroethyl)sulfinyl)ethyl)acrylamide),^10^ poly(*N*-(2-fluoroethyl)acrylamide),^11^ poly(*N*-(2,2-difluoroethyl)acrylamide),^12^ or various statistical copolymers of fluorinated monomers with hydrophilic monomers,^13−16^ show promising diagnostic potential for their excellent ^19^F relaxation properties and biocompatibility. However, their fluorine content is rather limited because increasing fluorine functionalization hampers polymer solubility in water and its magnetic relaxation properties.^13^ Therefore, the fluoropolymer structure must be generally optimized for the maximal ^19^F MR signal while retaining favorable physical and biological characteristics.

As alternative tracers, self-assembled amphiphilic block copolymer nanoparticles containing a fluorinated core-forming block provide high fluorine loadings.^17,18^ However, the high fluorine density within the nanoparticle core may reduce chain segment mobility and, consequently, significantly attenuate the ^19^F MRI signal. Nevertheless, this effect is known to be governed by the structure, hydration, and chain flexibility of the fluorinated block. For example, fluorine-rich diblock copolymers of thermoresponsive poly(*N*-(2,2-difluoroethyl)acrylamide) (PDFEAM) retain excellent ^19^F relaxation properties in water even after self-assembly upon heating above the lower critical solution temperature of PDFEAM (∼22 °C).^19^ This ability to retain ^19^F relaxation properties can be explained by the excellent hydration of the acrylamide-based polymer backbone even in the phase-separated state. For this reason, considering its permanent hydrophobicity, we selected poly(*N*-(2,2,2-trifluoroethyl)acrylamide) as a hydrophobic block for the present study.

Polymerization-induced self-assembly (PISA) has recently emerged as a powerful and straightforward method for synthesizing self-assembled polymer nanoparticles.^20−25^ In aqueous PISA, a water-soluble precursor block is chain extended by a hydrophobic block through direct polymerization in water, resulting in chain growth and block-copolymer self-assembly in a single step. Depending on the core-forming monomer solubility, PISA can be performed by dispersion or emulsion polymerization. In aqueous dispersion PISA, the core-forming monomers are soluble in the polymerization solvent (water), but their growing polymers become insoluble at a specific critical degree of polymerization (DP), leading to self-assembly during polymerization. Throughout PISA, the unreacted monomer is encapsulated into freshly formed nanoparticles, diffuses close to the growing chain end, and solvates the growing block, significantly boosting the polymerization rate. As such, PISA has numerous advantages over traditional methods, such as combining the synthesis and self-assembly process in a single step, shortening polymerization times, and requiring no purification. Moreover, polymer nanoparticles can be generally prepared at very high concentrations (>30 wt %) and in a wide range of morphologies. Using water as a polymerization solvent also creates an environmentally friendly path to aqueous nanoparticle dispersions. The library of PISA core-forming monomers may be still rather small, but more than a limitation this is an exciting challenge in polymer science.

In this context, the synthesis of ^19^F MRI nanotracers by PISA is also a promising direction with a high potential for the scalable development of advanced diagnostics. Thus far, studies on PISA of fluorinated monomers have relied on either emulsion PISA in water, which provides a low control over the polymerization process and limited access to advanced nanoparticle morphologies,^26,27^ or dispersion PISA in non-aqueous solvents (*i.e.*, alcohols),^28−30^ which requires an additional step of dialysis or ultracentrifugation to transfer the nanoparticles into an aqueous environment. Whittaker and colleagues reported the synthesis of ^19^F MRI nanotracers by dispersion PISA of styrene in isopropanol followed by extensive dialysis to switch the dispersant to water.^31^ The fluorine atoms were part of the hydrophilic shell, resulting in excellent ^19^F MR relaxivity, but the fluorine content was relatively low (<3 wt %), possibly limiting potential applications of such systems. To the best of our knowledge, no study on aqueous dispersion PISA of fluorinated monomers has been published to date.

Herein, we report the synthesis of ^19^F MRI nanotracers by aqueous dispersion PISA of *N*-(2,2,2-trifluoroethyl)acrylamide stabilized by a polyethylene glycol (PEG) hydrophilic block. After optimizing the reaction conditions to achieve full monomer conversion in a short time, we characterized the resulting diblock copolymer nanoparticles by NMR, size exclusion chromatography (SEC), dynamic light scattering (DLS), and transmission electron microscopy (TEM and CryoTEM). Subsequently, we thoroughly studied and optimized the ^19^F MR properties of the nanoparticles at 1.5 T and 4.7 T to create a robust platform for efficient diagnostic imaging. To demonstrate these properties, we performed a pilot *in vivo* study, where we subcutaneously administered the nanotracer to a healthy animal and monitored its distribution by ^19^F MRI.

## 2 Experimental Section

### 2.1 Materials

All chemicals, including *N,N*′-dicyclohexylcarbodiimide (DCC) and 4-(dimethylamino)pyridine (DMAP), were purchased from Sigma-Aldrich unless stated otherwise. *N*-Hydroxyethyl acrylamide (HEAM) was filtered through a short pad of basic alumina before being used to remove the inhibitor. Polyethylene glycol monomethyl ether, (PEG_91_-OH, 4 kDa), 2,2′-azobis[2-(2-imidazolin-2-yl)propane]dichloride (VA-044) were purchased from TCI. 2-(*n*-Butyltrithiocarbonate) propionic acid (BTPA)^32^ and *N*-(2,2,2-trifluoroethyl)acrylamide (TFEAM)^33^ were synthesized according to literature protocols. Water was deionized with a Millipore Milli-Q water purification system.

### 2.2 Synthesis of PEG-BTPA macroCTA

BTPA (238 mg, 1 mmol), PEG_91_-OH (2 g, 0.5 mmol), and DMAP (2.4 mg, 20 μmol) were dissolved in dry dichloromethane (DCM, 20 mL). After the reaction was cooled to 4 °C, a solution of DCC (206 mg, 1 mmol) in DCM (10 mL) was added dropwise. The reaction was allowed to warm to room temperature and stirred overnight, followed by the concentration under reduced pressure and precipitation in ice-cold diethyl ether. The precipitate was filtered and dried under reduced pressure. The crude polymer was purified by gel filtration on a Sephadex LH-20 column using methanol as an eluent. The polymer-containing fractions were collected and evaporated under reduced pressure to obtain the macro CTA as a yellow powder in a 62% yield. The chain-end modification was confirmed by MALDI-TOF (Figure S1). The absence of free BTPA in the polymer sample was confirmed by SEC by UV–vis detection at 320 nm. The esterification efficiency was determined by ^1^H NMR spectroscopy from the integral ratios of peaks at 4.20 and 3.25 ppm and was found to be 98.6%.

### 2.3 Synthesis of Fluorinated Nanoparticles by Aqueous Dispersion PISA of TFEAM

Fluorinated block copolymer nanoparticles were synthesized by RAFT-mediated dispersion PISA of TFEAM in water. In a typical experiment for the synthesis of PEG_91_-*b*-PTFEAM_100_ (total solid content, 6 wt %), TFEAM (70 mg, 0.459 mmol), PEG-BTPA (19 mg, 4.6 μmol), VA-044 {0.3 mg, 1 μmol, [CTA]_0_/[VA-044]_0_ = 4:1}, and 1,3,5-trioxane (5 mg) internal standard were dissolved in distilled water (1.4 mL), purged with nitrogen gas, and stirred in an aluminum heating block at 50 °C for 3 h (for PTFEAM DP 50–300), respectively, and 5 h (DP 400–600). The reaction was quenched by exposure to air followed by ^1^H and ^19^F NMR analysis. Monomer conversion was determined by ^1^H NMR spectroscopy of the reaction mixture upon dilution with CD_3_OD by comparing the residual vinyl peaks at 5.5–6.5 ppm with the signal of the internal standard. To calculate the ratio of both blocks, the nanoparticles were freeze-dried, dissolved in CD_3_OD, and analyzed by ^1^H NMR. To improve the ^19^F MRI potential of the nanoparticles, the same protocol was used to prepare another series of copolymers by PISA of a mixture of TFEAM and HEAM (molar ratios outlined in Table 2).

**Table 1:** Characteristics of PEG_91_-*b*-PTFEAM_*x*_ Block Copolymer Nanoparticles Synthesized by Aqueous Dispersion PISA of TFEAM with a PEG-BTPA Macro-CTA^a^

polymer	DP_T_^b^	Conv.^c^ (%)	F cont.^c^ (wt %)	*M*_n_^NMR,^^c^ (kg mol^–1^)	*M*_n_^SEC,^^d^ (kg mol^–1^)	*Đ*^d^	*D*_h_ (nm)/PDI^e^
F1	50	>99	23.8	11.9	25.2	1.14	173/0.509
F2	100	>99	29.1	19.6	41.7	1.13	63/0.180
F3	200	>99	32.7	34.9	75.6	1.19	94/0.096
F4	300	>99	34.1	50.2	90.2	1.21	97/0.083
F5	400	>99	34.8	65.5	130.5	1.31	136/0.026
F6	500	98	35.3	79.2	156.0	1.32	221/0.047

a All experiments were performed at 50 °C in water at a total solids content of 6 w/w % and [PEG-BTPA]_0_/[VA-044]_0_ = 4.

b PTFEAM target DP defined as the ratio [PTFEAM]_0_/[PEG-BTPA]_0_.

c Determined by ^1^H NMR.

d Determined by SEC against PMMA calibration.

e Determined by DLS in water at *c*_pol_ = 1 mg mL^–1^.

**Table 2:** Characteristics of the PEG_91_-*b*-[PTFEAM_*x*_-*stat*-PHEAM_*y*_] Block Copolymers and Their Nanoparticles Synthesized by Aqueous Dispersion PISA of TFEAM and HEAM as Core-Forming Monomers^a^

polymer	*f*_HEAM_^b^	DP_T_^c^	Conv.^d^ (%)	F cont.^d^ (wt %)	*M*_n_^NMR,^^d^ (kg mol^–1^)	*M*_n_^SEC,^^e^ (kg mol^–1^)	*Đ*^e^	*D*_h_ (nm)/PDI^f^
F2	0	100	>99	29.1	19.6	41.7	1.13	63/0.180
F2H1	0.1	100	>99	26.2	19.2	45.1	1.14	57/0.136
F2H2	0.2	100	>99	23.3	18.8	43.6	1.17	57/0.261
F2H3	0.3	100	>99	20.4	18.4	41.9	1.12	80/0.304
F3H2	0.2	200	>99	26.1	29.1	76.4	1.24	74/0.113
F4H2	0.2	300	>99	27.3	43.6	80.7	1.24	78/0.127
F5H2	0.2	400	>98	27.8	58.2	148.3	1.29	144/0.110

a All experiments were performed at 50 °C in water at a total solids content of 6 w/w % and [PEG-BTPA]_0_/[VA-044]_0_ = 4.

b Molar content of HEAM in the polymerization mixture.

c Target DP of the core-forming block.

d Determined by ^1^H NMR.

e Determined by SEC against PMMA calibration.

f Determined by DLS in water at *c*_pol_ = 1 mg mL^–1^.

### 2.4 Polymer Characterization

SEC was used to determine the molecular weights (*M*_w_—weight-averaged molecular weight and *M*_n_—number-averaged molecular weight) and dispersity (*Đ* = *M*_w_/*M*_n_) of the polymers on a Watrex Streamline system equipped with a Streamline P1 Pump, a Streamline AS2 Autosampler, a Streamline CT Column Thermostat, a Streamline UV detector, and a Streamline RI detector. The separation was performed on two PLgel 5 μm mixed-D columns in a series thermostatted at 55 °C in *N*,*N*-dimethylacetamide (DMA) containing 50 mM of LiCl at an elution rate of 0.5 mL min^–1^. Molar masses and dispersities were calculated against narrow dispersity poly(methyl methacrylate) standards.

Nuclear magnetic resonance (NMR) spectra were recorded on a Bruker Advance MSL 400 MHz NMR spectrometer at 25 °C in CD_3_OD, DMSO-*d*_6_ or a mixture of H_2_O/D_2_O (95/5 v/v). Unless otherwise stated, all ^19^F NMR spectra were measured at *c*_pol_ = 30 mg mL^–1^ using 20 μs pulse width, relaxation delay 8 s, acquisition time 1.5 s, and 64 scans, expressing all chemical shifts as ppm. The NMR spectra were processed using MestReNova 14.1 software, and the signal-to-noise ratios (SNRs) were calculated using the built-in MestReNova function. The *M*_n,NMR_ values were calculated by comparing the integral areas of peaks at 3.45 and 2.0 ppm.

Matrix-assisted laser desorption/ionization-time of flight mass spectrometry (MALDI-TOF MS) was performed on a Microflex LT MALDI-TOF MS (Bruker Daltonics) mass spectrometer. All mass spectra were recorded at an accelerating potential of 20 kV in the positive ion mode and in reflectron mode {matrix: 2-[3-(4-*tert*-butylphenyl)-2-methyl-2-propenylidene] malononitrile (DCTB)}. Samples were applied to the MALDI plate using the dried drop method. All measurements were calibrated using methoxy poly(ethylene glycol) (PEG, *M*_n_ = 2000 Da).

Critical micelle concentration (CMC) of nanoparticles was determined by fluorescence spectrometry on a Fluorolog FL 3-22 fluorimeter (Horiba Jobin Yvon, France) after Nile red (NR) encapsulation. A stock solution of NR in methanol (5 μL) was added to each sample of a series of aqueous nanoparticle dispersions (2 mL) of different polymer concentrations to a final NR concentration of 10^–6^ M. After incubation for 72 h at room temperature, fluorescence was measured using an excitation wavelength λ_ex_ = 550 nm. The CMC value was determined as the range at which the emission maximum of NR shifts from ∼620 nm (encapsulated NR) to ∼660 nm (free NR).

DLS measurements were used to determine the hydrodynamic diameters of the polymers in distilled water on a ZEN3600 Zetasizer Nano-ZS zeta potential analyzer (Malvern Instruments, UK). The polymer samples (*c*_pol_ = 1 mg mL^–1^) were filtered through a 0.22 μm PTFE syringe filter before measuring. The apparent Z-averaged hydrodynamic diameter of the particles, *D*_h_, was determined at a scattering angle of θ = 173°, and the DTS (Nano) program was used to evaluate the data.

TEM and cryo TEM (CryoTEM) observations were performed through a Tecnai G2 Spirit Twin 120 kV TEM (FEI), equipped with cryo-attachment (Gatan, cryo specimen holder) using a bright field imaging mode at an accelerating voltage of 120 kV. Aqueous solutions of the nanoparticles (3 μL, *c*_pol_ = 1 mg mL^–1^) were dropped on a copper TEM grid coated with a thin electron transparent carbon film. Before use, the grids were treated by glow discharge (Expanded Plasma Cleaner; Harrick Plasma, USA) to hydrophilize the carbon surface. After 2 min, the excess solution was removed by touching the bottom of the grids with filtering paper to minimize oversaturation during the drying process. Additionally, the samples were negatively stained with the uranyl acetate (2 μL of 1 wt %) solution dropped onto the dried nanoparticles and removed after 30 s as described above. Lastly, the samples were left to dry completely at room temperature before observation. The CryoTEM method employed different sample preparation. Aqueous solution of nanoparticles (4 μL) was deposited on a microscopy grid covered with lacey carbon supporting films (Agae Scientific) after hydrophilization by glow discharge (performed like in the previous method). The solution excess was removed by blotting (Whatman no. 1 filter paper) for ∼1 s and then the grids were immediately plunged into liquid ethane held at −183 °C. The vitrified samples were transferred into the microscope and observed at −175 °C under the conditions described above (120 kV, bright field mode)

### 2.5 Magnetic Resonance Properties

Relaxometry was used to measure the ^19^F relaxation times of fluorinated nanoparticles in distilled water on a 1.5 T Minispec 60 MHz relaxometer (Bruker Biospin, Germany) at 37 °C equipped with a fluorine probe (resonance frequency for fluorine was 54 MHz). The *T*_1_ relaxation times were measured with the inversion recovery sequence [repetition time (TR) = 0.1–10,000 ms, recycle delay = 4 s, scans = 4, echo time (TE) = 0.05 ms, monoexponential fitting, 16 points per fitting]. The *T*_2_ relaxation times were measured with the Carr–Purcell–Meiboom–Gill (CPMG) sequence (TR = 10,000 ms, recycle delay = 2 s, scans = 8, TE = 0.05 ms, monoexponential fitting, 20,000 points per fitting).

### 2.6 Imaging

^1^H/^19^F MR properties of fluorinated nanoparticles were measured in water by ^19^F MR spectroscopy (MRS) and ^19^F MR imaging (MRI) on a 4.7 T (Bruker Biospec 47/20, Ettlingen, Germany) scanner at 25 °C both in aqueous phantoms (i) and *in vivo* (ii). The MR scanner was equipped with a ^1^H/^19^F custom-made radiofrequency surface coil.^34^ (i) The *T*_2_-weighted ^1^H MR images were acquired for reference using a Rapid Acquisition with a Relaxation Enhancement (RARE) sequence with the following parameters: TR = 3000 ms, TE = 12 ms, effective echo time TE_eff_ = 36 ms, turbo factor = 8, bandwidth = 34,722 Hz, spatial resolution = 0.137 × 0.137 mm^2^, slice thickness = 0.85 mm, number of acquisitions NA = 1, and scan time = 1 min 12 s. The fluorine phantom MRI experiment was performed using two different sequences: (1) RARE sequence was used to measure samples with different HEAM contents, with the following parameters: TR = 2000 ms, TE = 5.60 ms, TE_eff_ = 22.40 ms, turbo factor = 10, bandwidth = 34,722 Hz, spatial resolution = 0.625 × 0.625 mm^2^, slice thickness = 9 mm, NA = 10–2000, and scan time = 2 m–6 h 40 min. (2) Multi-slice multi-echo sequence was used to measure F5H2 at different concentrations, with the following parameters: TR = 2000 ms, TE = 6.14, bandwidth = 34,722 Hz, spatial resolution = 0.779 × 0.779 mm^2^, slice thickness = 9 mm, NA = 1–100, and scan time = 4 m–7 h. The imaging experiment was performed at 25 °C in 0.5 mL Eppendorf tube phantoms containing various concentrations of nanoparticles (*c*_pol_ = 5–30 mg mL^–1^) with the cross sections of the tubes shown in the phantom images. The ^19^F image was overlapped with the anatomic ^1^H image with spin-echo-based contrast. (ii) An *in vivo* measurement was performed using one healthy female BALB/c mouse as a proof of principle. The mouse was anesthetized with 5% isoflurane (Baxter, Deerfield, USA) for induction and 1.5–0.5% isoflurane for maintenance. The respiratory rate was monitored throughout the study using a trigger unit (Rapid Biomedical, Berlin, Germany). To avoid eye dryness and its potential damage, an eye cream (Ophtalmo-Septonex, Zentiva, Czech Republic) was applied before the measurement. Subcutaneous injection of F5H2 (*V* = 200 μL, *c*_pol_ = 60 mg mL^–1^) in phosphate-buffered saline (PBS) was applied into the inner side of the right hind leg. On the side of the radiofrequency coil, we put the Eppendorf tube containing F5H2 (*V* = 200 μL, *c*_pol_ = 60 mg mL^–1^) in PBS as a reference. For the ^1^H reference image, we used the same sequence parameters as in the phantom measurement. ^19^F MRS single-pulse sequence (TR = 2000 ms, bandwidth = 200 ppm, NA = 150, and ST = 5 min) was used to confirm the presence of the fluorine signal and to fine-tune the resonance frequency. For ^19^F MR imaging, we performed spectrally resolved MRI using a chemical shift imaging (CSI) sequence with the following parameters: TR = 200 ms, bandwidth = 40 ppm, spatial resolution = 2.81 × 2.83 mm^2^, slice thickness = 10 mm, and ST = 13 min 40 s. ^19^F-MR CSI and spectroscopic data were processed and analyzed using custom MATLAB (https://mathworks.com, Matlab R2021b, The MathWorks, Inc., USA) scripts.

All animal protocols were approved by the Ethics Committee of the Institute for Clinical and Experimental Medicine and the Ministry of Health of the Czech Republic (no. 58/2014) in accordance with the European Communities Council Directive (2010/63/EU).

### 2.7 Cytotoxicity of Fluorinated Nanoparticles

Human primary cells and culture conditions—human primary dermal fibroblasts (HFs) were kindly gifted by the Institute of Experimental Medicine, Academy of Sciences of the Czech Republic. The cells were cultured in Dulbecco’s modified Eagle’s medium supplemented with 10% fetal bovine serum and 1% penicillin–streptomycin (100 U mL^–1^, Thermo Fisher Scientific, USA) at 37 °C under a humidified 5% CO_2_ atmosphere. HFs were subcultured every four days and used for experiments up to the n passage.

Cytotoxicity assay—HFs were seeded at a concentration of 8 × 10^3^/100 μL/well on 96-well plates and cultured for 24 h at 37 °C in an incubator. Subsequently, the cells were treated with F5H2 nanoparticles in a twofold serial dilution starting from the highest final concentration (2 × 10^3^ μg mL^–1^). After 72 h of incubation, 10 μL of PrestoBlue cell viability reagent (Thermo Fisher Scientific, USA) were added into the medium with cells in the presence of F5H2 nanoparticles and into the blank wells (medium with nanoparticles without cells) and incubated for 4 h 30 min at 37 °C. The fluorescence intensity of the developed dye (resorufin) was measured at an Ex/Em wavelength of 550/590 nm (bandwidth 20 nm) in the top-optic mode on a Spark multimode microplate reader (Tecan Group Ltd., Männedorf, Switzerland). The relative cell viability (%) after exposure to fluorinated nanoparticles was expressed as a percentage of viable cells after the treatment in comparison with the control set to 100%. The experiment was performed three times in sextuplicates.

## 3 Results and Discussion

**Scheme 1:**
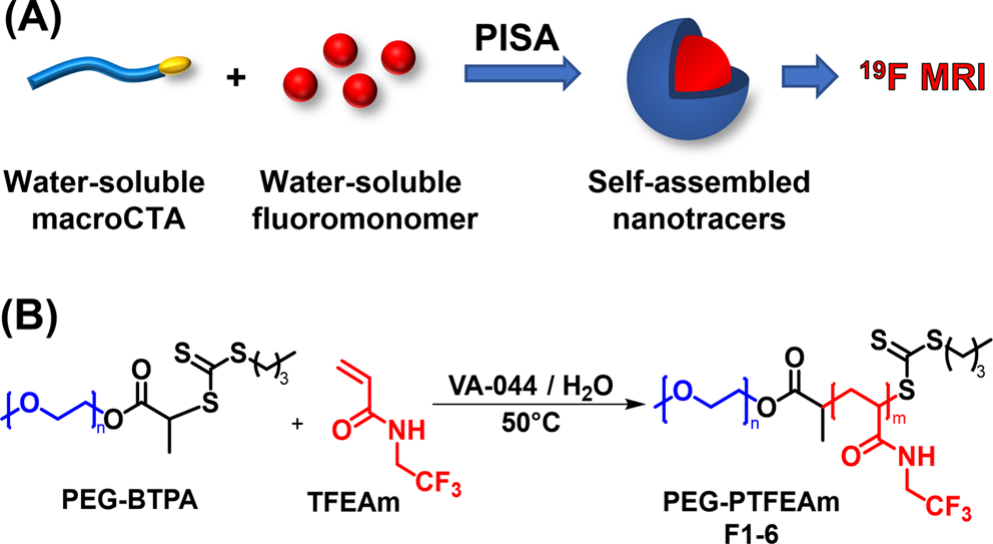
Synthesis of ^19^F MRI Tracers by RAFT-Mediated Aqueous Dispersion PISA of TFEAM: (A) Schematic Illustration and (B) Reaction Scheme

*N*-(2,2,2-Trifluoroethyl)acrylamide (TFEAM) was selected as a core-forming monomer to synthesize ^19^F MRI nanotracers by aqueous dispersion PISA for its solubility in water and favorable ^19^F MR properties. Although most semifluorinated monomers, including *N*-(2,2,2-trifluoroethyl)acrylate and *N*-(2,2,2-trifluoroethyl)methacrylate, are disqualified for their negligible solubility in water, acrylamide-containing TFEAM is soluble (estimated as 7 wt % at 50 °C) enough for aqueous dispersion PISA. Furthermore, TFEAM contains three magnetically equivalent fluorine atoms and thus provides a sole singlet in its ^19^F NMR spectrum. Lastly, this monomer can be easily prepared in high quantities by straightforward acrylation of trifluoroethylamine, as shown in Scheme 1.

RAFT-mediated aqueous dispersion PISA of TFEAM was performed in distilled water using 4 kDa PEG_91_-BTPA as a macromolecular chain-transfer agent and VA-044 as a water-soluble initiator at a total solid concentration of 6 wt %. Even though PISA has been reported for much higher monomer concentrations, the targeted nanoparticle concentration sufficed for the intended application as a ^19^F MRI tracer. The polymerizations were performed at 50 °C as higher reaction temperatures (60 °C) led to macroscopic precipitation of the reaction mixture for higher target DPs (>150). Such behavior is commonly observed in aqueous PISA when using PEG-based macroCTAs, which tend to aggregate in water at elevated temperatures, as reported by Armes and colleagues.^21,35^

**Figure 1:**
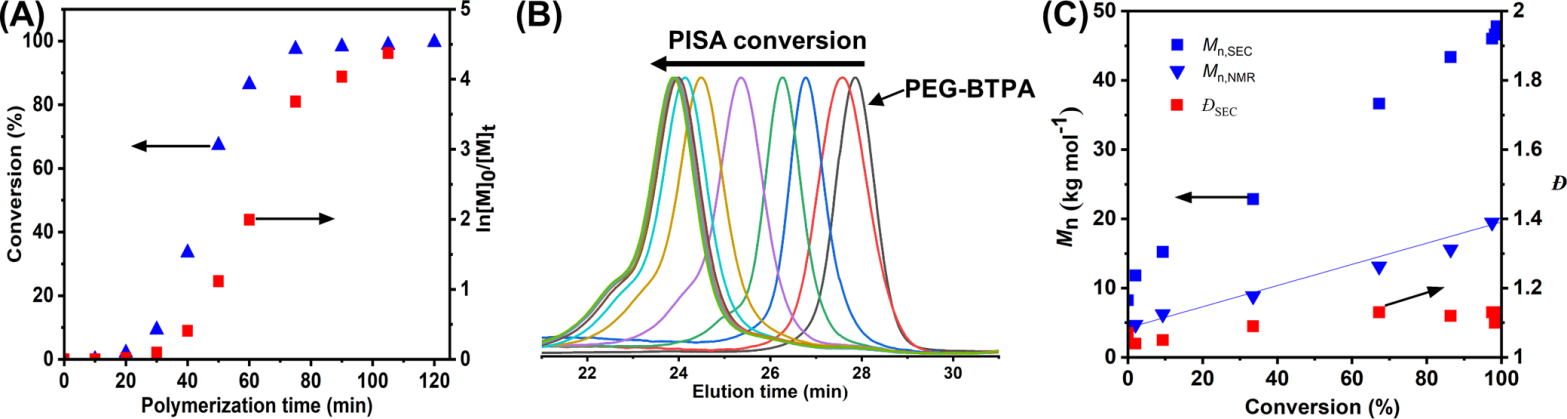
Aqueous dispersion PISA kinetics of TFEAM at 50 °C using PEG-BTPA macroCTA {[TFEAM]_0_:[PEG-BTPA]_0_ = 100:1}: (A) variation of monomer consumption as a function of polymerization time, (B) evolution of SEC traces during polymerization eluted with DMA/LiCl, and (C) variation of experimental number-average molar mass (*M*_n_) and dispersity (*D̵*) as a function of conversion of TFEAM determined by SEC against PMMA calibration (squares), respectively NMR (triangles). The blue line represents the theoretical *M*_n_ value.

The polymerization kinetics measurement of DP_PTFEAM_ 100 and 200 copolymers revealed a controlled polymerization process (Figures 1 and S2, S3). After an initial induction period (20 min for target DP 100), with a slow rate, polymerization significantly accelerated at 40 min (for DP 100), and the solution became opalescent, indicating the formation of self-assembled nanoparticles. Following polymerization kinetics, SEC showed that all polymerizations proceeded in a controlled way, with a linear increase of polymer molar mass with conversion. The SEC-based molar masses were systematically higher than the corresponding theoretical values due to the differences in the hydrodynamic sizes of our copolymers and poly(methyl methacrylate) (PMMA) SEC standards in *N*,*N*-dimethylacetamide (DMA) eluents. On the other hand, the NMR-based molar masses fit the theoretical values. Throughout the polymerization, the molar mass dispersities remained low (*D̵* < 1.13), which is typical for dispersion PISA. The observed high-molecular-weight shoulders in chromatograms most probably originate from the end-to-end coupling of living macroradicals.

**Figure 2:**
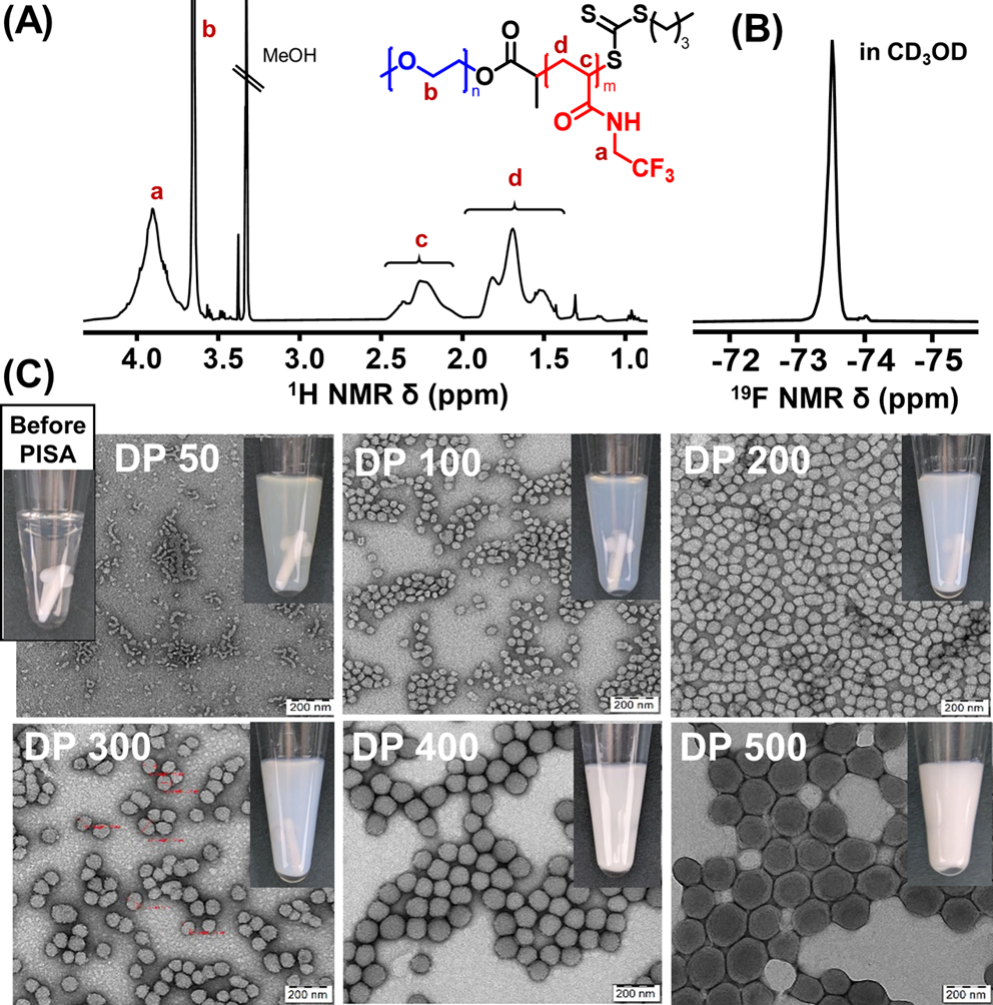
Representative ^1^H (A) and ^19^F (B) NMR spectra of PEG_91_-*b*-PTFEAM_100_ (F2) in CD_3_OD at 400 MHz and (C) transmission electron micrographs of F1–F6 nanoparticle dispersion; the scale bars represent 200 nm. Insets: physical appearance of as-prepared nanoparticle dispersions in water.

The optimized PISA protocol was applied to synthesize a series of fluorinated copolymer nanoparticles differing in PTFEAM block length (F1-6, DP = 50–500, Table 1 and Figure 2). Longer polymerization times (3–5 h) were necessary to ensure full monomer conversions aiming to directly use the fluorinated nanoparticle dispersions as ^19^F MRI tracers without any purification. During the polymerization, all copolymers up to DP 500 formed nanoscale colloid dispersions without any signs of macroscopic aggregation or precipitation.

The copolymer compositions were determined by ^1^H NMR after freeze-drying and dissolving in deuterated methanol and were very close to the initial monomer/macroCTA feed ratios. ^19^F NMR spectroscopy in CD_3_OD revealed a sharp singlet at −73.5 ppm corresponding to the −CF_3_ side-chain groups. SEC analysis confirmed that copolymer chain length increased with the TFEAM/macroCTA feed ratio, whereas dispersity remained reasonably low (*Đ* < 1.35) (Table 1 and Figure S4A).

The hydrodynamic diameters of the nanoparticles were measured upon dilution in water by DLS, (Figure S4B). Except for the shortest DP 50 copolymer, nanoparticle size gradually increased with chain length from 63 nm (F2, DP 100) to 221 nm (F5, DP 500), with narrow size distributions (dispersity 0.03–0.18). Nanoparticle size and morphology were also studied by TEM (Figure 2), corroborating the DLS data. The results showed a mixed morphology consisting of small spheres with worm-like micelles in the shortest DP 50 nanoparticles, which can be ascribed to the lack of proper core stabilization. Spherical morphologies were observed in nanoparticles with higher DPs (100–500). The nanoparticle diameters were, nevertheless, much larger than the calculated extended copolymer lengths. For this reason, cryoTEM measurements were performed in F2 and F6, in water, to explore the possibility of vesicular morphology (Figure S5). CryoTEM of F2 highlighted irregular, mostly donut-shaped nanoparticles. On the other hand, F6 nanoparticles showed a homogeneous cryoTEM contrast, suggesting large compound micelles rather than hollow polymersomes.^36^

^19^F MR imaging of PEG-b-PTFEAM micelles in water unsurprisingly provided limited information due to the restricted mobility of the core-forming fluorinated segments, thus accounting for the poor magnetic relaxation properties of the fluorine nuclei. The main ^19^F NMR signal can be ascribed to the traces of unreacted monomer, which disappears after ultracentrifugation leaving barely any signal in the spectrum (Figure S6). This is in sharp contrast to the excellent ^19^F MR properties of the homologous thermoresponsive poly(*N*-(2,2-difluoroethyl)acrylamide) copolymers, which were retained even after copolymer self-assembly in water.^19^ Although PEG-b-PTFEAM nanoparticles lacking a ^19^F NMR signal may be useful for developing various stimuli-responsive “on–off” reporting systems, their core-forming block structure required optimization for the intended application as ^19^F MRI tracers.

**Figure 3:**
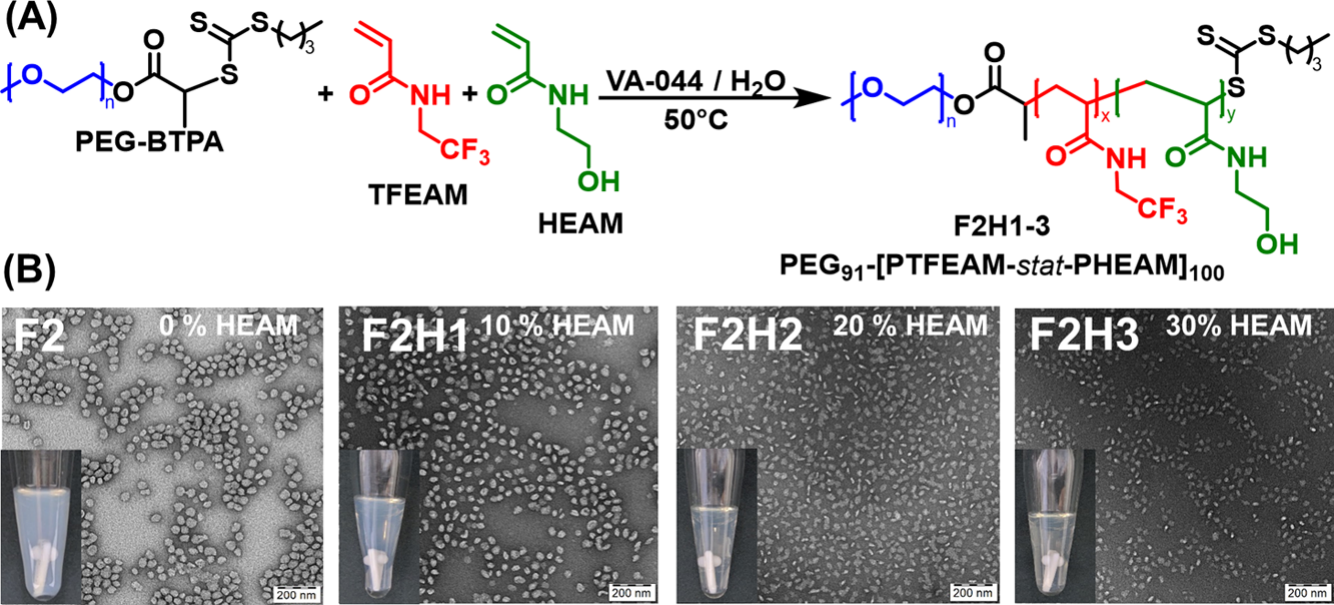
Synthesis of PEG_91_-*b*-[PTFEAM_*x*_-*stat*-PHEAM_*y*_] block copolymers with a core partly hydrophilized by aqueous PISA: (A) reaction scheme and (B) TEM images of nanoparticles differing in HEAM content (*F*_HEAM_ = 0–0.4) at constant core-forming block length (DP 100); the scale bars represent 200 nm. Insets: physical appearance of as-prepared nanoparticle dispersions in water.

To improve the ^19^F MRI tracing potential of our nanoparticles, we introduced a small part of the hydrophilic comonomer *N*-hydroxyethyl acrylamide (HEAM) into the PISA reaction mixture (Figure 3A), thereby diluting the local fluorine concentration in the core and improving chain hydration and mobility, ultimately enhancing the ^19^F NMR/MRI signals. Copolymers with different TFEAM/HEAM feed ratios (9:1 for F2H1, 8:2 for F2H2, and 7:3 for F2H3, respectively) and the same core DP 100 were synthesized and characterized using the aforementioned methods (Figure S7). In their ^1^H NMR spectra, the signals of HEAM side-chain protons overlapped with those of PEG (−CH_2_–OH, 3.65 ppm) and methanol (−NH–CH_2_–, 3.33 ppm). Therefore, the presence of HEAM was confirmed by ^1^H–^13^C HSQC 2D NMR (Figure S8), which separated the overlapped peaks by their ^13^C shifts.

The measured PHEAM content was close to that of the initial monomer feed. Despite the modification, the fluorine content remained very high (20 wt % for F2H3). Stable colloidal dispersions were obtained in water at HEAM contents up to 30%. Higher hydrophilic comonomer contents (>40%) resulted in the loss of amphiphilicity, as shown by DLS, where separate sub-10 nm copolymer coils prevailed. The size of the nanoparticles did not change substantially with the HEAM content. These findings were in line with TEM experiments (Figure 3B), where a change in nanoparticle morphology was observed with the increase in HEAM content from 50 nm “donuts” (0% HEAM) to irregular-shaped separated nanoparticles of the same size (F2H3). The morphology change was confirmed by cryogenic TEM microscopy (Figure S9) for nanoparticles F2H2 and F2H3, as well.

**Figure 4:**
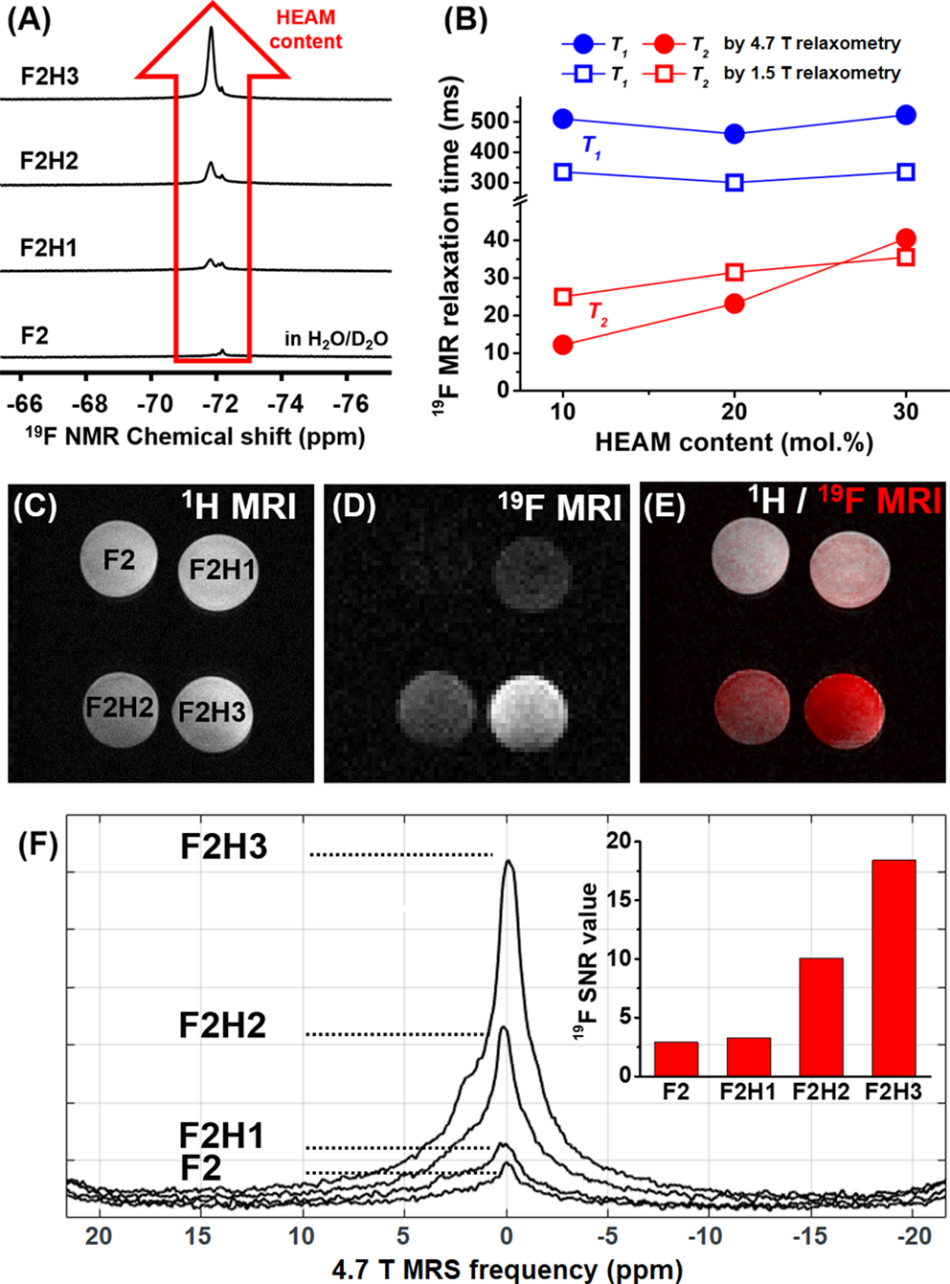
Partial core hydrophilization with HEAM enhances the ^19^F MR signal of fluorinated nanoparticles (*c*_pol_ = 30 mg mL^–1^, DP_core_ = 100) in aqueous solutions. (A) Evolution of nanoparticle ^19^F NMR spectra (400 MHz) as a function of HEAM content; (B) variation of ^19^F MR relaxation times as a function of HEAM content; (C) ^1^H and (D) ^19^F RARE MRI of nanoparticles (4.7 T) with different HEAM contents; (E) overlay image of ^19^F MRI (red) and ^1^H MRI (gray); and (F) ^19^F MR spectra (4.7 T) of nanoparticles centered and used for MRI acquisition; inset: comparison of ^19^F MRI SNR values.

Our ^19^F MR measurements confirmed the strong impact of the HEAM comonomer on the ^19^F MR properties of fluorinated nanoparticles. The HEAM-free nanoparticles F2 showed virtually no ^19^F NMR signal due to the limited relaxation of fluorine atoms. Moreover, the main signal may be ascribed to traces of unreacted monomers (−72.2 ppm) encapsulated within the micelle core, as suggested by the broad peak distribution (Figure 4A).

Although the TFEAM monomer is detected only in trace amounts (<0.1 mol %), its superior ^19^F relaxation to that of the self-assembled fluoropolymer led to its dominant NMR signal. The identity of this peak was confirmed by ^19^F NMR measurements of the nanoparticle dispersion after the addition of additional TFEAM, which only increased peak intensity, without any change in chemical shifts. Conversely, the disassembly of the same nanoparticle dispersion by deuterated methanol led to a sharp polymer peak in the ^19^F spectrum, strongly exceeding the trace monomer peak, due to enhanced polymer relaxation in methanol and high fluorine content.

In aqueous solutions, the nanoparticles showed a strong ^19^F NMR signal only after HEAM incorporation. The nanoparticle ^19^F NMR signal increased rapidly with the HEAM content despite the slight decrease in fluorine content (from 29 to 20 wt %). The SNRs of the nanoparticle dispersions increased from 52 (F2H1) to 307 (F2H3) due to the enhanced magnetic relaxation of fluorinated segments. To demonstrate that the strong ^19^F NMR signal resulted from the polymer nanoparticle and not from the potential residual encapsulated monomer, F2H3 micelles were freeze-dried and purified from any low-molar-mass impurities by gel filtration in methanol, followed by re-assembly by nanoprecipitation. The ^19^F NMR spectrum of purified micelles was nearly identical to the spectrum of diluted as-obtained micelles (Figure S10).

The ^19^F MR relaxations of nanoparticles F2 and F2H1-3 were studied in water by MR relaxometry at both 1.5 T and 4.7 T (Figure 4B and Table S1). In general, high-intensity ^19^F MR images require short spin–lattice *T*_1_ relaxation times and long spin–spin *T*_2_ relaxation times.^4^ While excessively long *T*_1_ relaxation times may result in inevitably long acquisition times, very short *T*_2_ times could limit the imaging to non-standard sequences with extremely short echo times (TEs), which are not routinely implemented in MR scanners.

The *T*_1_ relaxation times were measured with a single-pulse sequence using monoexponential fitting, showing reasonably low values (*T*_1_ = 350–500 ms), without significant differences between polymers. In turn, the *T*_2_ relaxation times were measured with the CPMG sequence at 4.7 T and showed a significant increase with the HEAM comonomer content from 12.2 ms (F2H1) to 40.5 ms (F2H3). Accordingly, the increase in the ^19^F MR signal strength with the HEAM content may be ascribed to enhanced *T*_2_ relaxation times. Even though these *T*_2_ values are lower than those commonly observed in water-soluble semifluorinated polymers,^13^ they suffice to achieve a strong ^19^F MR signal for effective imaging thanks to the high fluorine content (>20 wt %).

The favorable ^19^F MR relaxation parameters of HEAM-rich copolymers allowed us to visualize nanoparticles *in vitro* in Eppendorf tube phantoms by 4.7 T ^19^F MRI using a conventional Rapid Imaging with Refocused Echoes (RARE) pulse sequence with long echo times (TE = 5.6 ms, Figures 4C–F and S12). The ^19^F MRI SNR of nanoparticle tracers significantly increased with the HEAM content, providing us with outstanding tracing sensitivity and with the ability to shorten the acquisition times necessary for reliable visualization. Therefore, the copolymers with a high HEAM content (F2H2 and F2H3) may be then relevant for potential *in vivo* tracing applications.

Micellar stability is one of the key characteristics of self-assembled nanopharmaceuticals, strongly affecting their pharmacological profile and the release of potentially encapsulated drugs. Hence, we studied the equilibrium stability of the nanoparticles by measuring their CMC, which expresses the concentration threshold above which the micelles are formed. When diluted below their CMC, the micelles disassemble into individual unimer chains. Herein, the CMC of the fluorinated nanoparticles were measured in water after encapsulating Nile red as a solvatochromic fluorescence probe (Figure S11).

The HEAM-free F2 nanoparticles (PEG_91_-*b*-PTFEAM_100_) showed high equilibrium stability, with CMC values ranging from 2 to 4 mg L^–1^, corroborating the tight micelle core packing suggested above, which leads to a poor ^19^F NMR signal in water. Increasing the HEAM content enhanced the ^19^F MRI signal but impaired nanoparticle stability due to weakened core hydrophobic interactions. For example, F2H2 showed relatively high CMC values (125–250 mg L^–1^), which may be detrimental to potential biomedical applications given the expected rapid blood clearance. To foster micelle stability, we increased the DP of the core-forming block from 100 (F2H2) to 400 (F5H2) while maintaining the HEAM/TFEAM molar feed ratio constant at 2:8.^37^ As expected, such a chain extension had a positive effect on nanoparticle stability, and the CMC of F5H2 dropped to the range of 31–63 μg mL^–1^ suitable for applications. Moreover, the increased nanoparticle chain length did not attenuate the ^19^F NMR signal (Table S1), and the SNR values even increased from 106 (DP 100) to 286 (DP 400), reflecting the increasing fluorine content.

**Figure 5:**
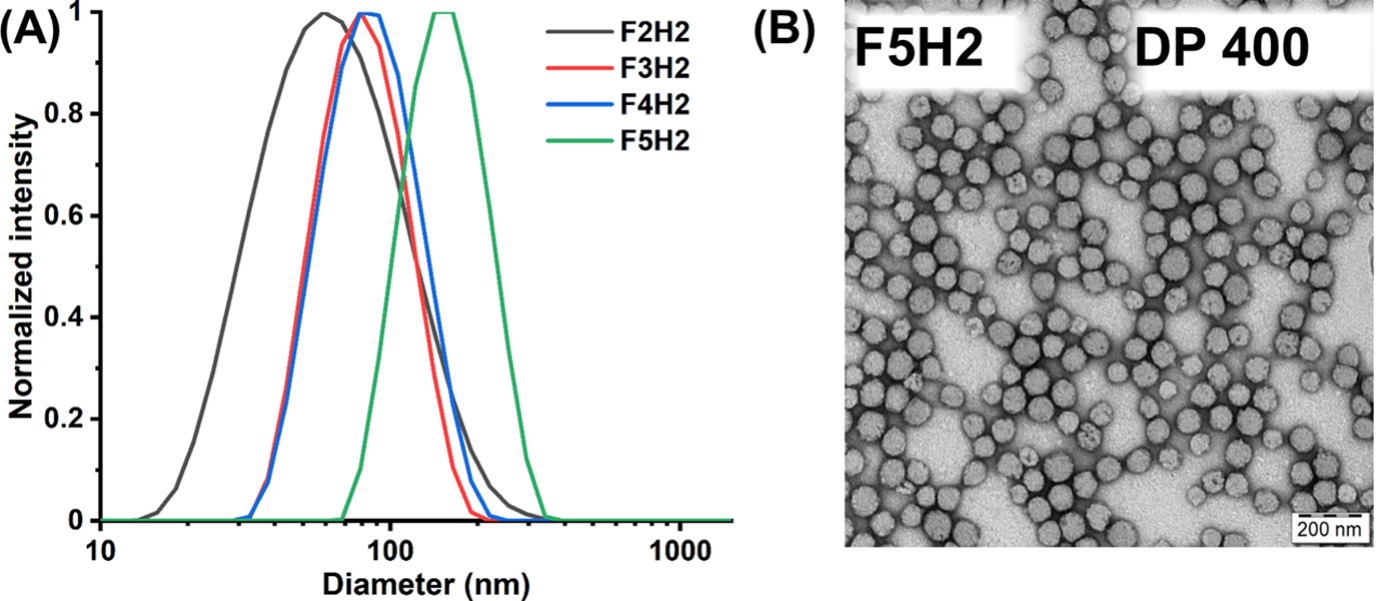
(A) Intensity-weighted DLS size distributions of PEG_91_-*b*-[PTFEAM_*x*_-*stat*-PHEAM_*y*_] copolymer nanoparticles (*x*/*y* = 8:2) differing in core-forming block DP (100–400) in water (*c*_pol_ = 1 mg mL^–1^) and (B) transmission electron micrograph of F5H2 nanoparticles (DP 400); the scale bar represents 200 nm.

The increase in core block length had a major impact on the nanoparticle size and morphology (Figures 5 and S14). The hydrodynamic diameter of the nanoparticles increased from 57 (F2H2, DP 100) to 144 (F5H2, DP 400) nm, which was accompanied by a morphological transition from irregular-shaped separated nanoparticles (DP 100) to polymersomes (DP 200 and 300) and large compound micelles (DP 400). This morphological transformation may be attributed to a change in the micelle core packing parameter. Based on their favorable stability and ^19^F NMR characteristics, the DP 400 nanoparticles (F5H2) were selected as an optimal ^19^F MRI tracer for further evaluation.

The aqueous dispersions of the optimized F5H2 nanoparticles showed favorable ^19^F MR relaxivity and MRI properties. The longitudinal relaxation time did not change significantly with the copolymer DP, remaining reasonably low (*T*_1_ = 469 ms for F5H2). Furthermore, the transverse relaxation time *T*_2_ even slightly increased to 62.5 ms for F5H2, in line with our previous ^19^F NMR observations. We believe that such an improvement in *T*_2_ as a function of the core DP is associated with the enhanced relaxation of fluorinated segments close to the core–shell interface of higher order nanoparticles.

**Figure 6:**
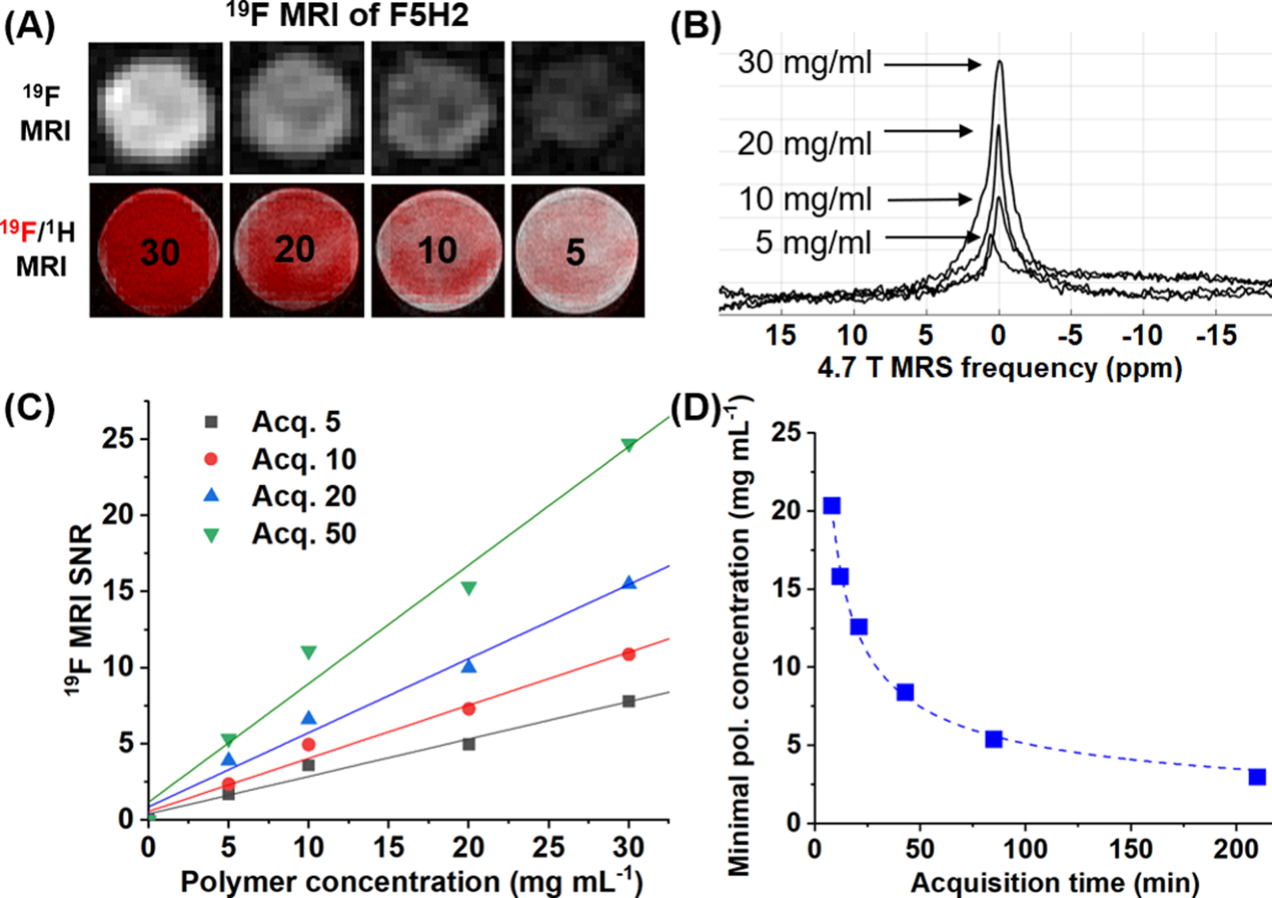
*In vitro*^19^F MRI/MRS properties of optimized F5H2 nanoparticles in water; (A) ^19^F (top), ^19^F (red) overlaid on ^1^H (colors in grayscale) RARE MRI (bottom) of F5H2 at different polymer concentrations (20 acquisition scans); the numbers 30, 20, 10, and 5 express *c*_pol_ as mg mL^–1^. (B) ^19^F MR spectra (4.7 T) centered and used for MRI acquisition; (C) variation of the ^19^F MRI SNR ratio as a function of F5H2 concentration at different numbers of acquisition scans; and (D) minimal polymer concentrations needed to reliably visualize (SNR = 3.5) the F5H2 nanotracer at different MRI acquisition times.

The superior ^19^F MR relaxation of F5H2 micelles is reflected in their significantly improved ^19^F MRI properties (Figures 6 and S15). The nanoparticles were successfully visualized *in vitro* using a 4.7 T instrument at different polymer concentrations and numbers of acquisition scans. The MRI SNRs increased linearly with the polymer concentration (Figure 6C). From this correlation, the minimum concentration needed to reliably visualize the nanoparticles (SNR = 3.5) can not only be calculated but also quantitatively expresses the MRI sensitivity of our nanotracer at specific acquisition parameters. Indeed, the minimal traceable concentration decreases with the increase in acquisition time (Figure 6D).

Before advancing to preliminary *in vivo* experiments, the non-cytotoxicity of F5H2 was confirmed by resazurin-based PrestoBlue cell viability assay upon incubation with human dermal fibroblast cells (HFs; Figure S16). After 72 h, no signs of toxicity were observed within the polymer concentration range tested (up to 2 mg mL^–1^). These results suggest the excellent cytocompatibility of the nanoparticles.

**Figure 7:**
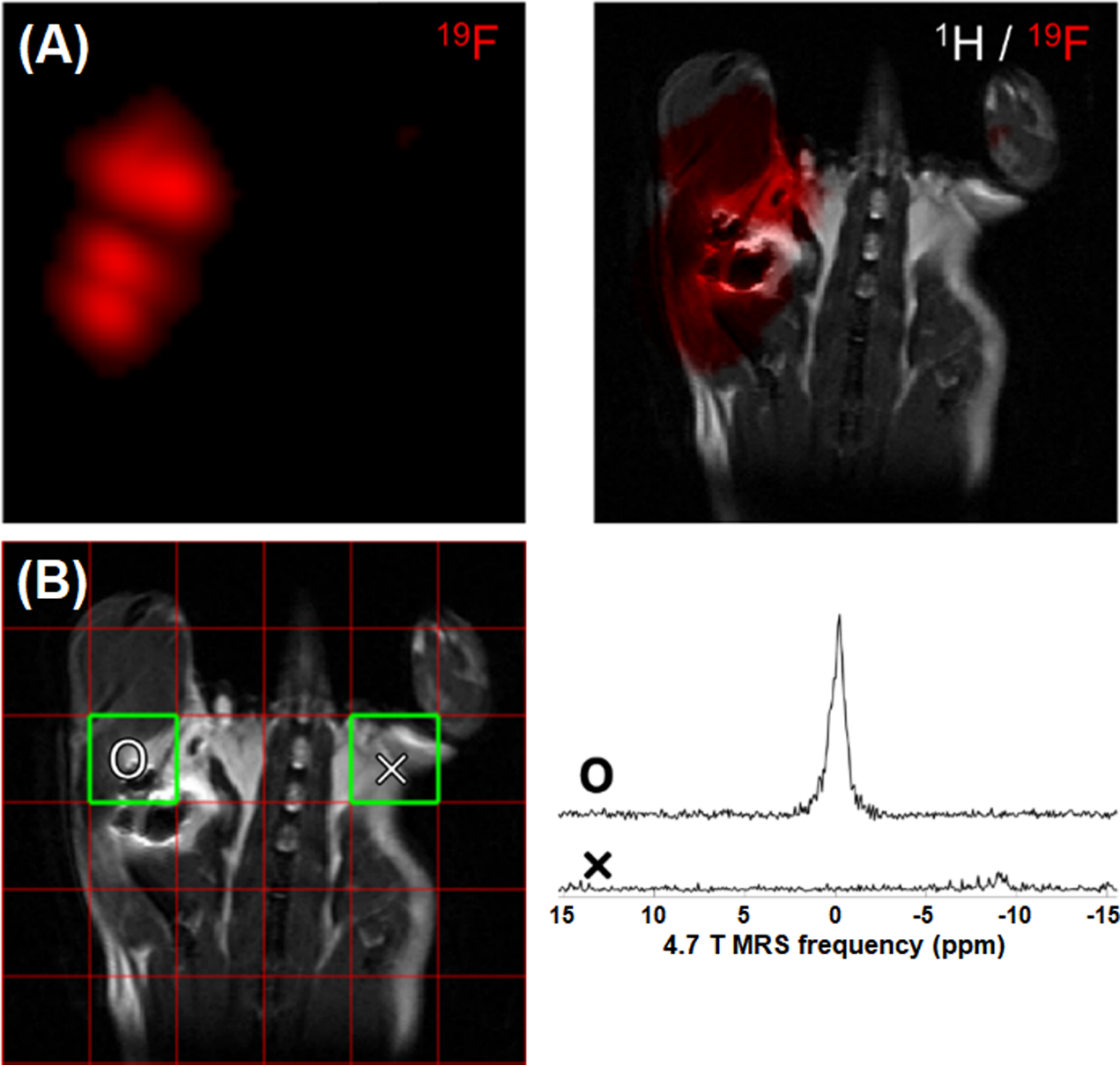
*In vivo*^19^F MRS and MRI of F5H2 nanotracer 2 h after subcutaneous injection into healthy BALB/c mouse; (A) coronal hot spot ^19^F MRI (left) and overlapped ^1^H/^19^F (right) images, where the F5H2 fluorine signal is highlighted in red color and (B) ^19^F MR CSI spectroscopic grid on a ^1^H MR reference scan (left); the red grid shows measured CSI voxels and the green squares represent the targeted anatomical area from which the corresponding summation spectra are displayed (right).

Given the promising results of the *in vitro* experiments, we performed *in vivo*^19^F MRS and MRI experiments as a proof-of-concept study (Figure 7). For *in vivo* imaging, acquisition times should be reasonably short to minimize the time that the animals spend under anesthesia and between measurements to monitor metabolic processes. We subcutaneously administered the F5H2 tracer in PBS (*V* = 200 μL, *c*_pol_ = 60 mg mL^–1^) into the inner side of the right hind leg of a healthy female BALB/c mouse. The target area was chosen primarily for ease of injection. In addition, the probe was only applied to the right leg, so the contralateral left leg of the mouse served as a control. At the edge of the surface coil, we placed an Eppendorf tube containing the same F5H2 solution (*V* = 200 μL) that we administered as a reference for the fluorine peak assignment.

After the injection, the non-localized ^19^F MR spectrum of the whole animal plus the reference tube showed three clearly distinguishable peaks–one corresponding to the polymer and two others assigned to the isoflurane anesthetic (Figure S17). The signal coming from the polymer was separated by a large chemical shift. After setting the precise Larmor frequency for the polymer measurements, we removed the F5H2 reference tube from the magnet without changing the position of the animal and repeated the spectroscopic measurement. The leftmost peak became significantly attenuated, whereas the other two peaks showed only negligible changes in intensity. Therefore, we confirmed that the leftmost peak (already with the resonance frequency centered at 0 ppm) reflects only the presence of the F5H2 polymer administered *in vivo*. The minor changes in the intensity of the remaining two peaks derive from the variable isoflurane concentration used to maintain the animal under anesthesia.

The ^19^F MR CSI sequence data show that the fluorine signal originates only from the injection region (Figure 7). Figure 7B shows the ^19^F MR CSI grid spectral view of a ^1^H MR image reference scan. The red grid displays CSI voxels, and the green box represents the area from which the corresponding summation of spectra is displayed. When comparing spectra from two boxes highlighted in green (left and right leg), 2 h after F5H2 administration, we confirmed the presence of F5H2 in the right leg only–the site of probe administration. No signal was detected in the area corresponding to the left leg. The high-resolution CSI matrix shows well the signal intensities of the applied polymer and corresponds to the CSI image reconstructed for the polymer frequency range only (Figure S18). Moreover, the F5H2 polymer provides a high SNR *in vivo* in both the spectra (SNR = 12.09) and images (SNR = 42.9) with short acquisition times (5 min for spectra, 13 min 40 s for images).

The preliminary *in vivo* data suggest the significant potential of our new fluorinated nanoparticles as nanotracers for medical purposes involving intramuscular or subcutaneous applications or as markers for imaging cells such as transplanted pancreatic islets labeled *in vitro*.^38^ More detailed *in vivo* experiments are, however, beyond the scope of this study, which focused on PISA synthesis and preliminary ^19^F MRI of fluorinated nanoparticles in phantoms but will nevertheless be conducted in the near future by our research group. Finally, given the current rapid progress in the area of MRI instrumentation, the MRI sensitivity of our nanotracers may be substantially amplified by increasing magnetic field strengths and using cryogenically cooled radiofrequency coils.

## 4 Conclusions

Fluorinated nanoparticles prepared by aqueous PISA of water-soluble *N*-(2,2,2-trifluoroethyl)acrylamide (TFEAM) using PEG-BTPA as a macroCTA show well-defined block copolymer architectures and size and morphology highly dependent on the core-forming block length. Despite their high fluorine content, their negligible ^19^F NMR signal in water results from the tight packing of the fluorinated hydrophobic core which leads to poor magnetic relaxation of the fluorinated segments and thus requires optimization. Increasing the hydrophilicity of their fluorinated core by incorporating a small amount of *N*-hydroxyethyl acrylamide (HEAM) significantly improves the ^19^F MRI signal of the nanoparticles. These nanotracers display good ^19^F MR relaxation properties and ^19^F MRI performance. However, micelle core hydrophilization leads to morphological changes and decreases micelle stability. Nevertheless, increasing the core-forming block length while maintaining the optimized TFEAM/HEAM ratio unchanged improves the micelle stability. Such optimized HEAM-containing nanoparticles with a lengthened core-forming block perform well as ^19^F MRI tracers, showing even better ^19^F MR relaxation properties and MRI sensitivity than low-DP copolymers on a 4.7 T instrument with magnetic fields close to those used in clinical imaging. Although the MRI sensitivity of our tracer is slightly lower than that of previously reported water-soluble semifluorinated linear polymers, it can be applied for *in vivo* tracing applications involving high local concentrations of nanotracers (*e.g.*, subcutaneous administration). A more detailed *in vivo* study is currently underway, and the results will be published soon in a separate article. Ultimately, our nanoparticles may be used as hydrophobic drug delivery carriers combining both tracing and therapy in the same system (*i.e.*, theranostics). Considering their straightforward synthesis, highly modular properties, and a broad range of potential applications, these polymer nanoparticles stand out as excellent materials for advanced biomedical research.

## Supporting Information Available

Characterization of synthesized polymers by MALDI-TOF, NMR, SED, Cryo-TEM, and MR (PDF)
