# Supplementary material for: Synthesis of 19F MRI Nanotracers by Dispersion Polymerization-Induced Self-Assembly of N-(2,2,2-Trifluoroethyl)acrylamide in Water
Source: Biomacromolecules. 2022 Oct 17;23(11):4814–24. doi: 10.1021/acs.biomac.2c00981 (PMC10797588; doi:10.1021/acs.biomac.2c00981)
Supplement: Supplementary file 1 — bm2c00981_si_001.pdf [file bm2c00981_si_001.pdf]

Supporting information

Synthesis of ^{19}F MRI nanotracers by dispersion polymerization-induced self-assembly of *N*-(2,2,2-trifluoroethyl)acrylamide in water

Vyshakh M. Panakkal,[a] Dominik Havlicek,[b,c] Ewa Pavlova,[d] Marcela Filipová,[d] Semira Bener,[a] Daniel Jirak[b,c] and Ondrej Sedlacek[a,*]

[a]Department of Physical and Macromolecular Chemistry, Faculty of Science, Charles University, 128 40 Prague 2, Czech Republic

[b]Department of Diagnostic and Interventional Radiology, Institute for Clinical and Experimental Medicine, 140 21 Prague, Czech Republic

[c]Faculty of Health Studies, Technical University of Liberec, Studentská 1402/2, Liberec 461 17, Czech Republic

[d]Institute of Macromolecular Chemistry, AS CR, 162 06 Prague 6, Czech Republic

E-mail: sedlacek@natur.cuni.cz

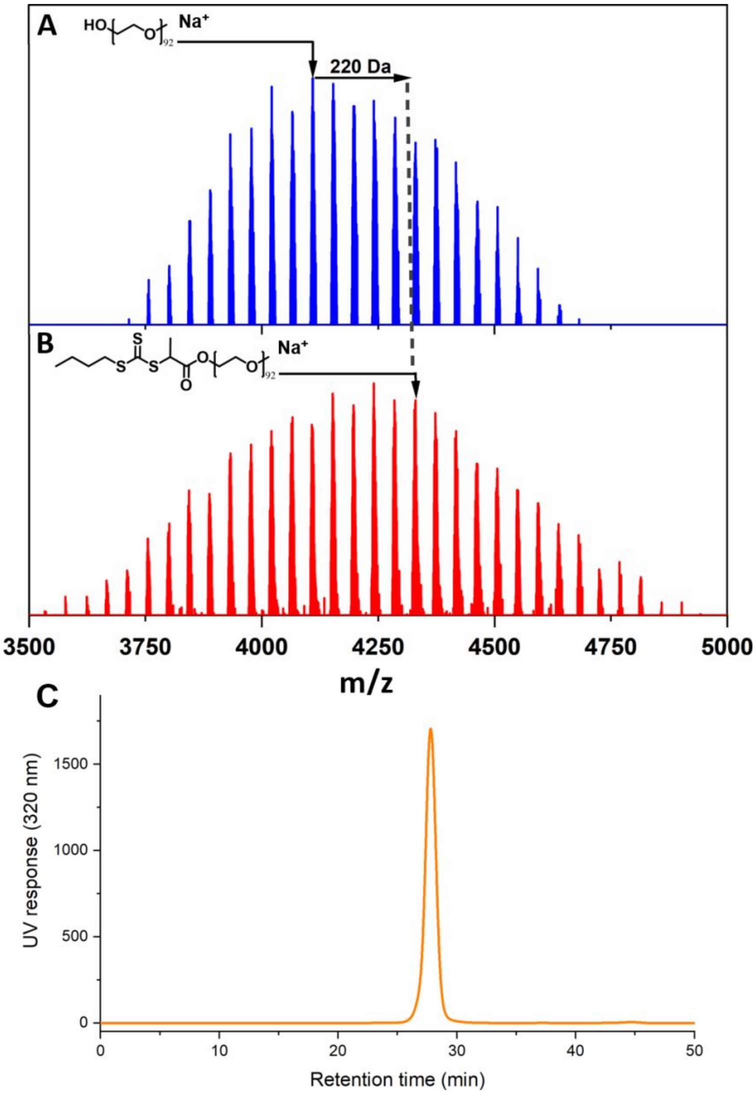

Figure S1. MALDI-TOF spectra of (A) PEG-OH and (B) PEG-BTPA. (C) SEC trace of PEG-BTPA with UV detection at 320 nm.

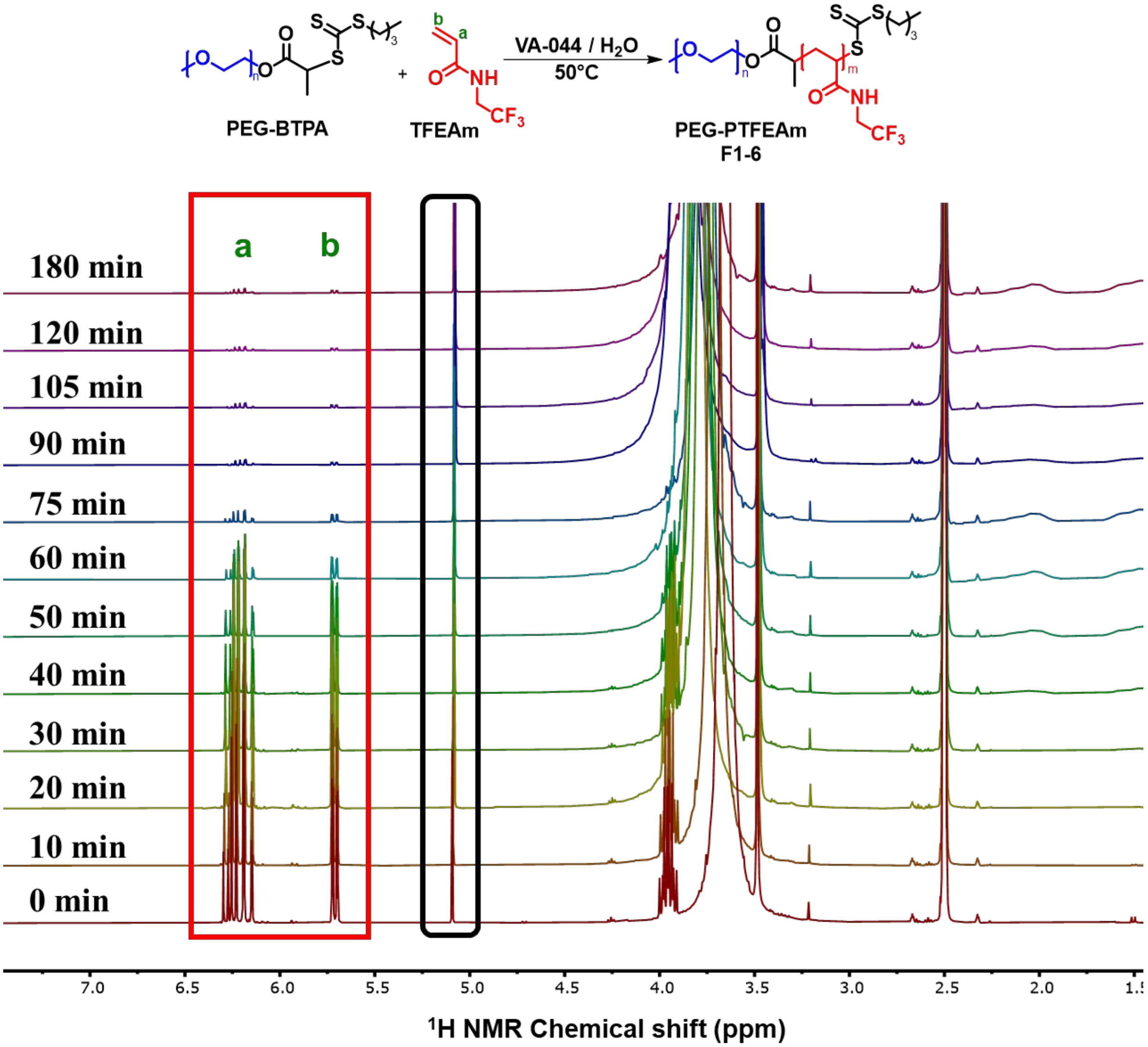

Figure S2. Aqueous dispersion PISA kinetics of TFEAM at 50°C using PEG-BTPA macroCTA ($[TFEAM]_0$:$[PEG\text{-}BTPA]_0$ = 100:1). Evolution of ^1H NMR spectra of the polymerization mixture measured upon dilution with DMSO-d6 with 1,3,5-trioxane (5.09 ppm) as an internal standard.

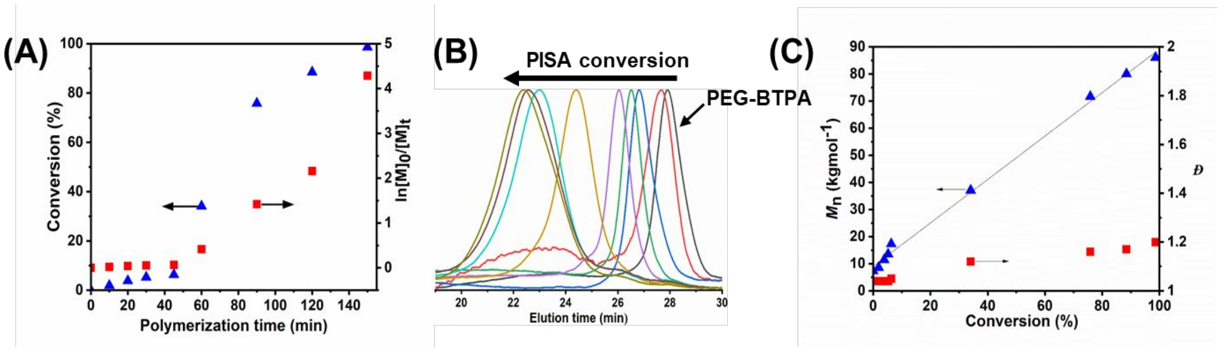

Figure S3. Aqueous dispersion PISA kinetics of TFEAM at 50°C using PEG-BTPA macroCTA ($[TFEAM]_0$:$[PEG\text{-}BTPA]_0$ = 200:1). (A) Consumption of monomer vs. time. (B) Evolution of SEC traces during the polymerization eluted with DMA/LiCl. (C) Experimental number-average molecular masses and dispersities plotted vs. conversion of TFEAM determined by SEC with PMMA calibration.

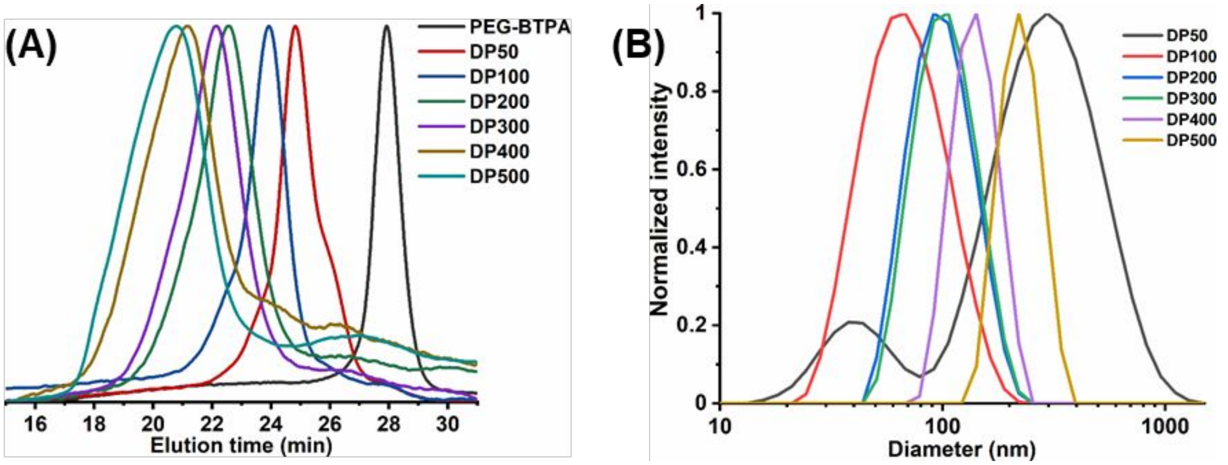

Figure S4. (A) SEC traces of PEG-b-PTFEAM copolymers (F1-F6) eluted with DMAc. (B) Intensity-weighted DLS size distributions of PEG-b-PTFEAM nanoparticles (F1-F6) in water (c_{pol} = 1 mg mL^{-1}).

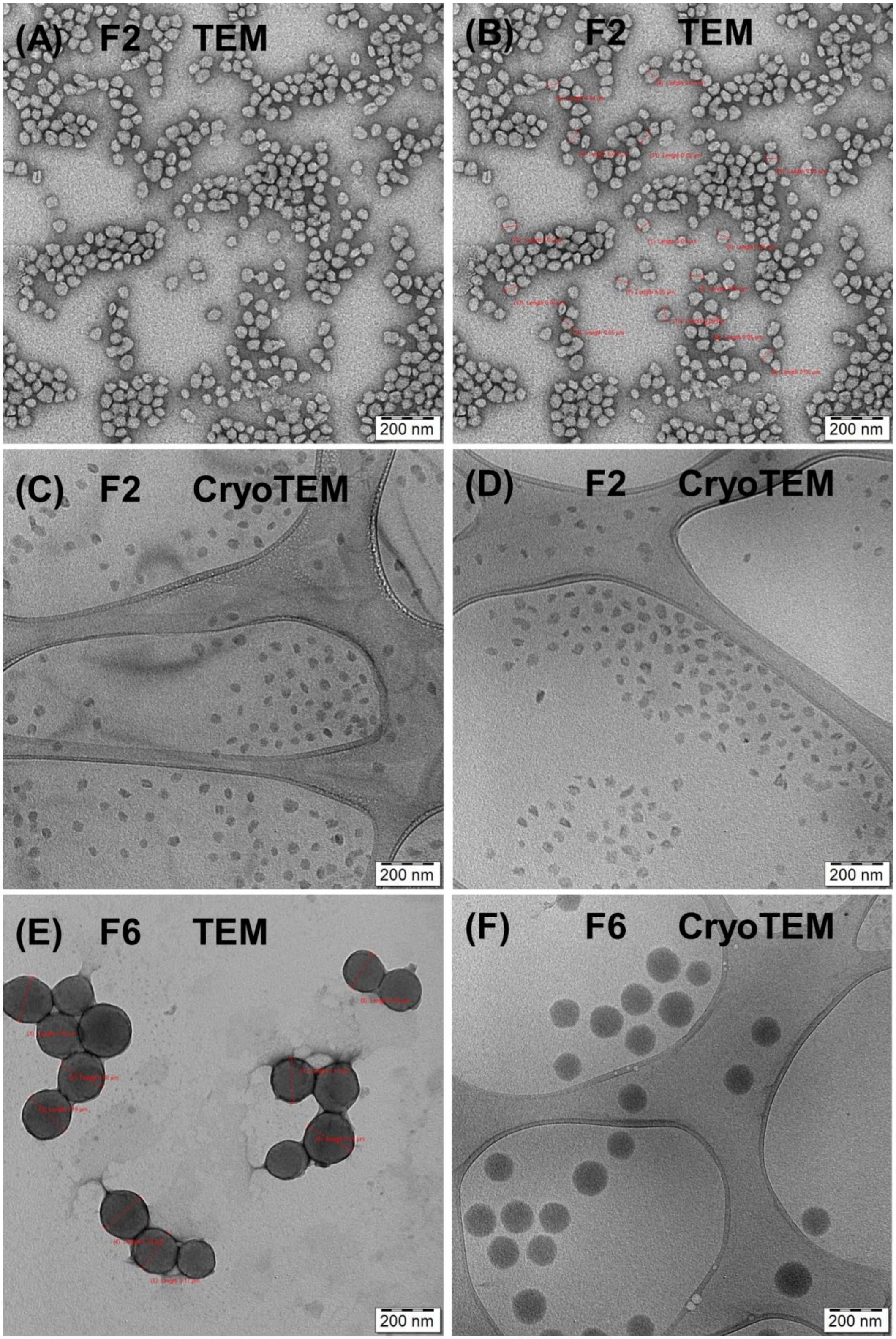

Figure S5. TEM (A, B, E) and Cryo-TEM (C, D, F) images of copolymers F2 (A, B, C, D) and F6 (E, F).

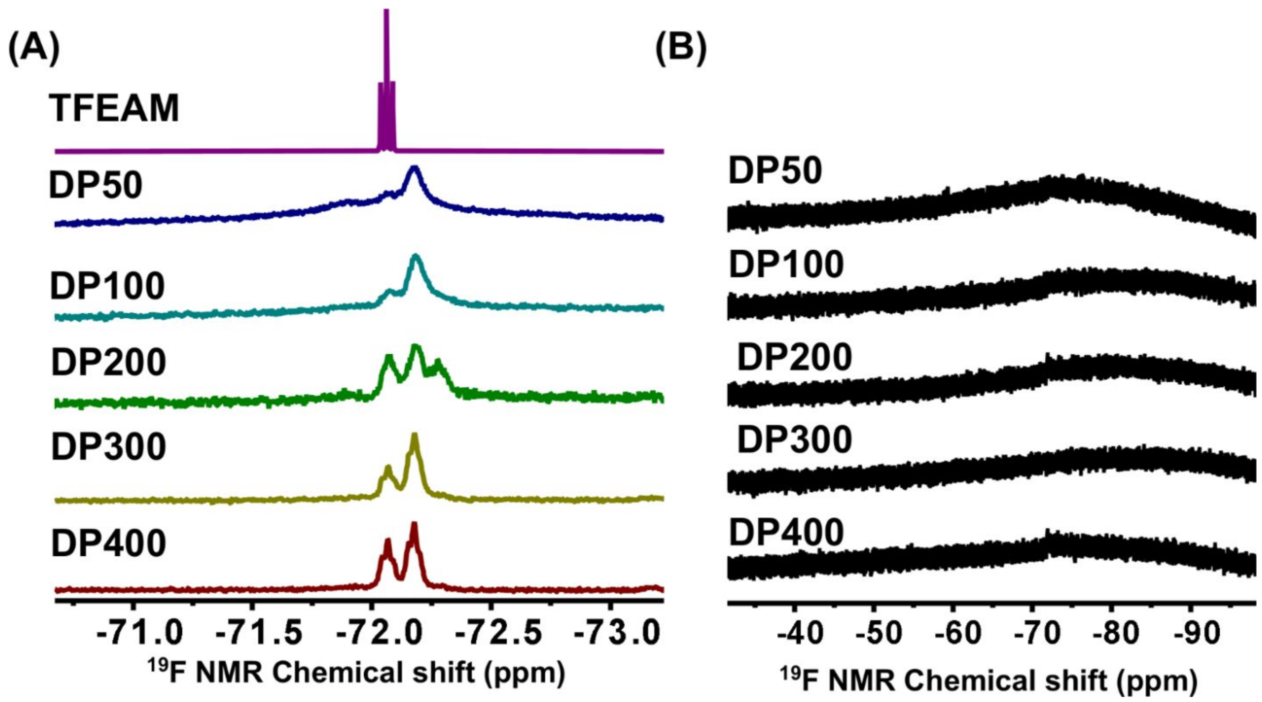

Figure S6. (A) ^{19}F NMR spectra of as-obtained F1-F5 (PEG_{91}-*b*-PTFEAM) dispersions in water/D_2O (95:5 v/v, c_{pol} = 20 mg mL^{-1}), (B) ^{19}F NMR spectra of F1-F5 (PEG_{91}-*b*-PTFEAM) in water/D_2O after ultracentrifugation.

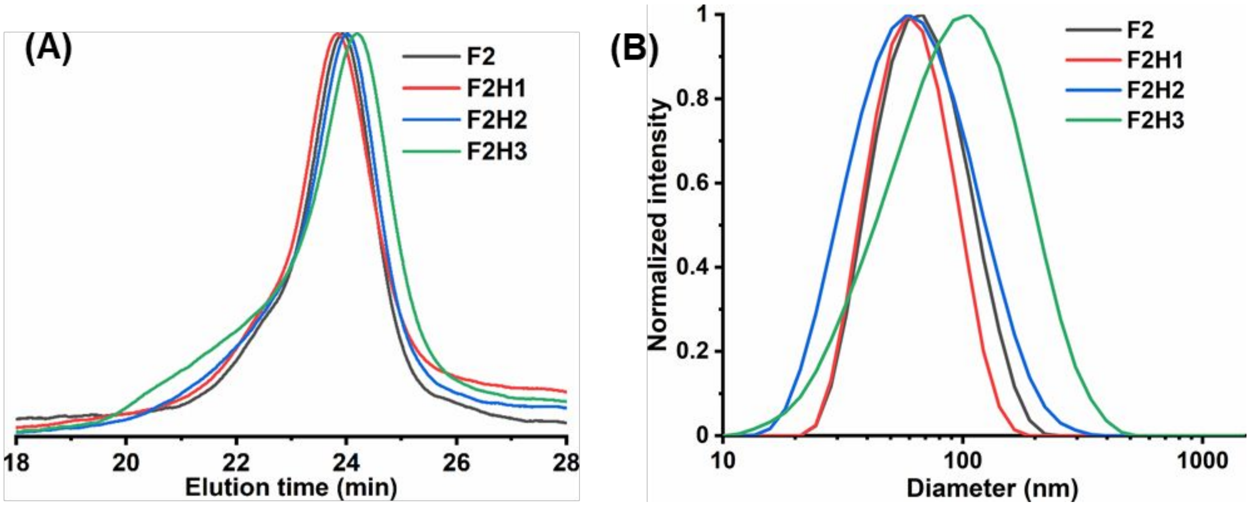

Figure S7. (A) SEC traces of copolymers F2, F2H1, F2H2, respectively F2H3 eluted with DMAc. (B) Intensity-weighted DLS size distributions of F2, F2H1, F2H2, respectively F2H3 nanoparticles in water (c_{pol} = 1 mg mL^{-1}).

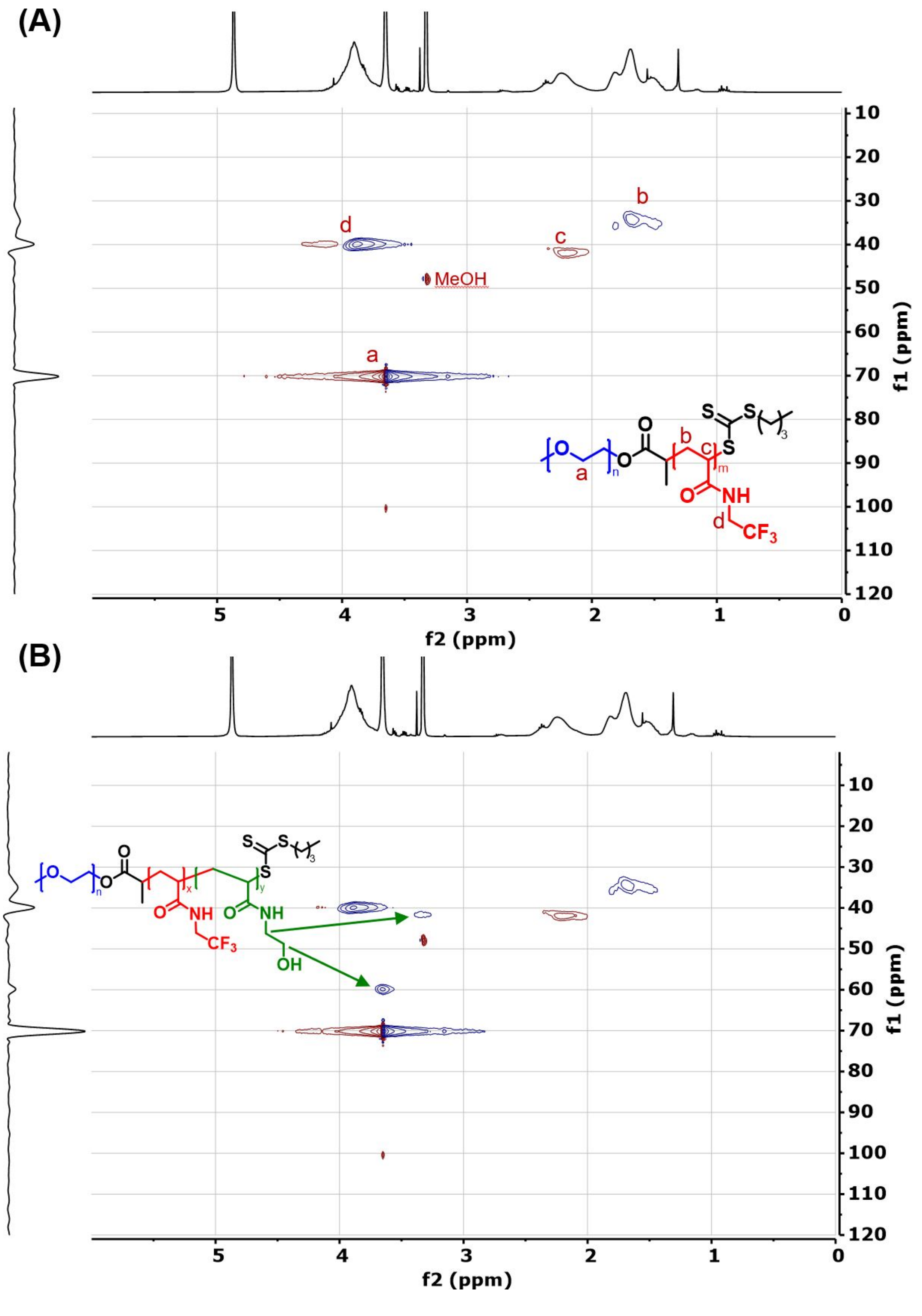

Figure S8. ¹H-13C HSQC spectra of F2 (A), respectively F2H2 (B) measured in CD_3OD.

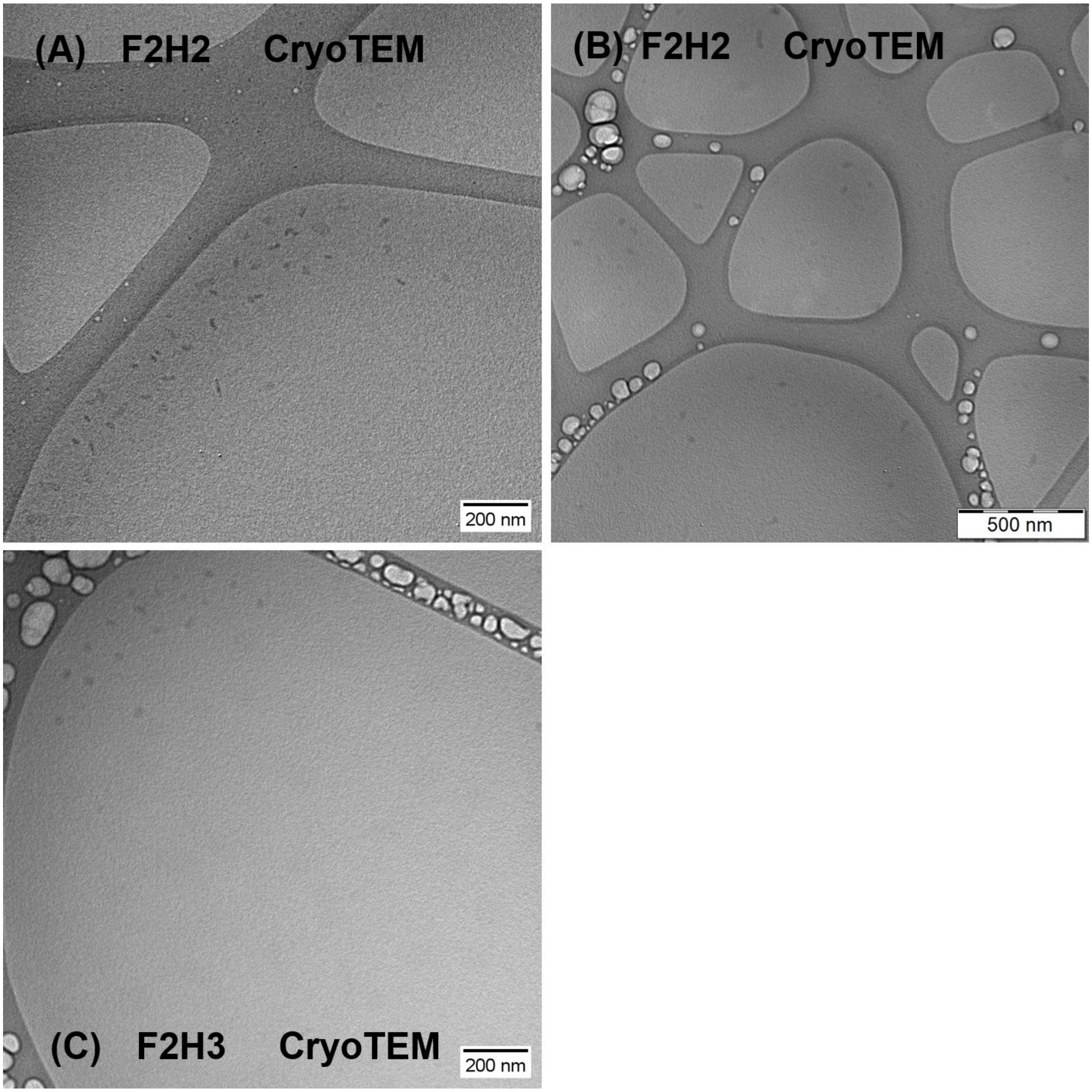

Figure S9. Cryo-TEM images of copolymers F2H2 (A, B) and F2H3 (C).

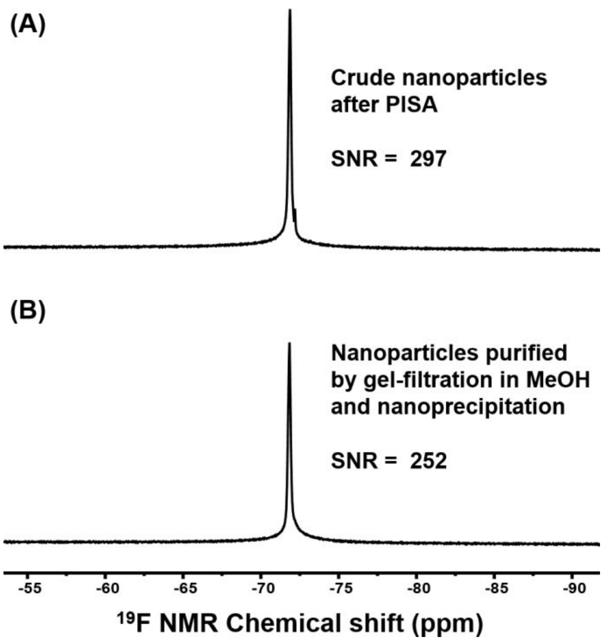

Figure S10. ^{19}F NMR spectra of F2H3 nanoparticles in water/D_2O (95:5 v/v, c_{pol} = 20 mg mL^{-1}). (A) Nanoparticles obtained by PISA, (B) Nanoparticles with removed monomer traces - obtained after PISA by freeze-frying, gel-filtration in MeOH and nanoprecipitation. SNR = ^{19}F NMR signal-to-noise ratio.

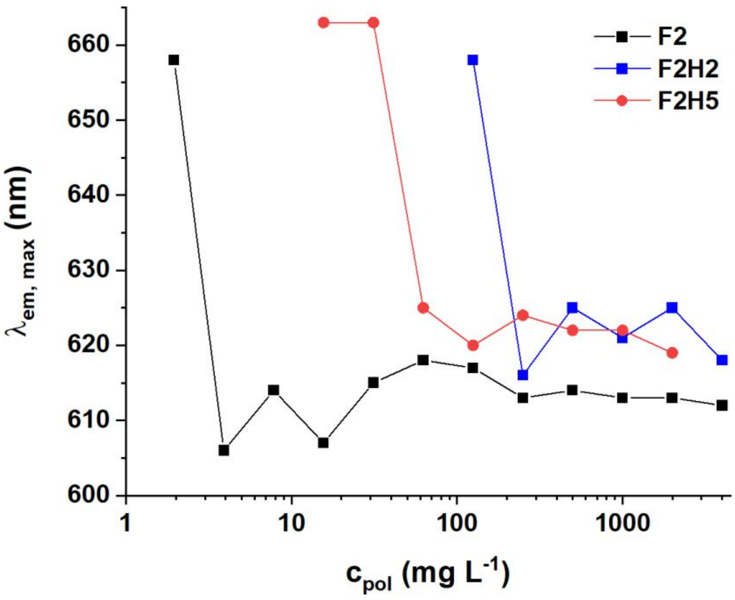

Figure S11. The dependence of maximum emission wavelength of Nile Red on the concentration of F2, F2H2, respectively F5H2 in water.

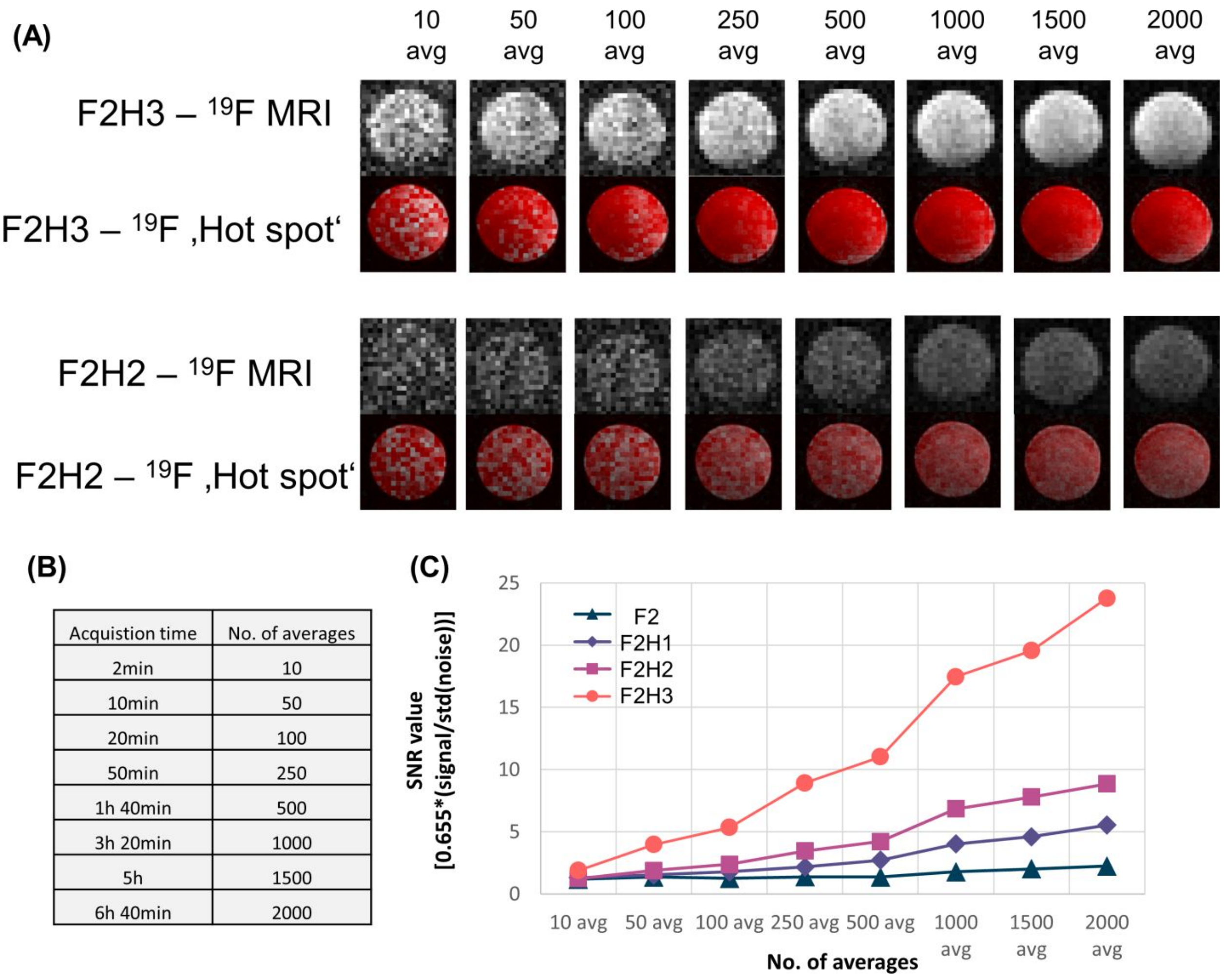

Acquistion time	No. of averages
2min	10
10min	50
20min	100
50min	250
1h 40min	500
3h 20min	1000
5h	1500
6h 40min	2000

Figure S12. (A) ^{19}F RARE and ^{1}H MRI images (4.7 T, without post-processing) of F2H3 and F2H2 nanoparticles in water at different number of acquisitions. Overlay images show a ^{19}F MRI signal in red color. (B) Overview of MRI acquisition times. (C) Dependance of ^{19}F MRI SNR on the number of acquisitions.

Table S1. ^{19}F MR characteristics of prepared PEG_{91}-b-[$PTFEAM_x$-*stat*-$PHEAM_y$]block copolymers in water.

Polymer	T_1[a] (ms)	T_2[a] (ms)	T_1[b] (ms)	T_2[b] (ms)	^{19}F NMR SNR[c]
F2H1	510	12.2	335	26	51.7
F2H2	461	23.2	300	25	106
F2H3	523	40.5	335	32	307
F5H2	469	62.5	n.d.[d]	n.d[d]	286

[a]Measured by 4.7 T MRI. [b]Measured by 1.5 T relaxometry. [c]Measured by 400 MHz NMR. [d]Not determined.

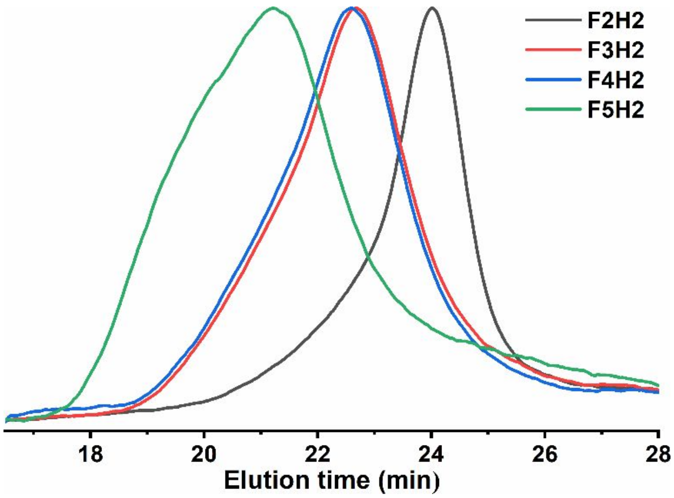

Figure S13. SEC traces of copolymers F2H2 (DP 100), F3H2 (DP 200), F4H2 (DP 300) respectively F5H2 (DP400) eluted with DMAc.

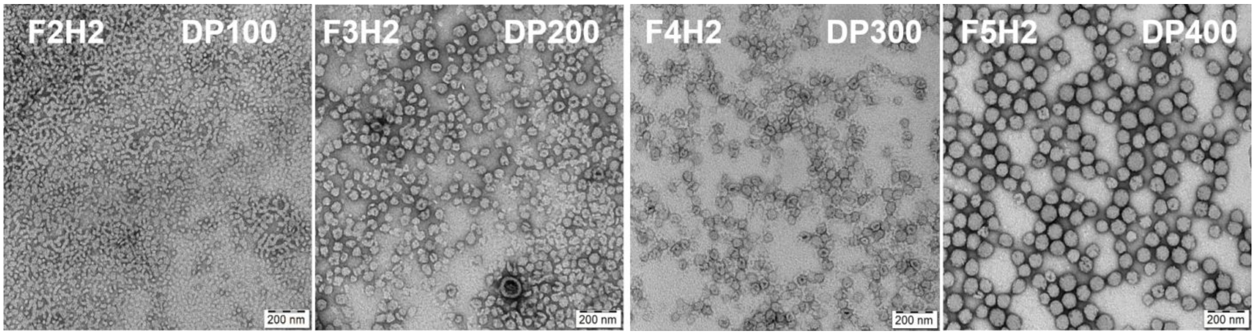

Figure S14. Transmission electron micrographs of F2H2, F3H2, F4H2 and F5H2 nanoparticle dispersions. Scale bars represent 200 nm.

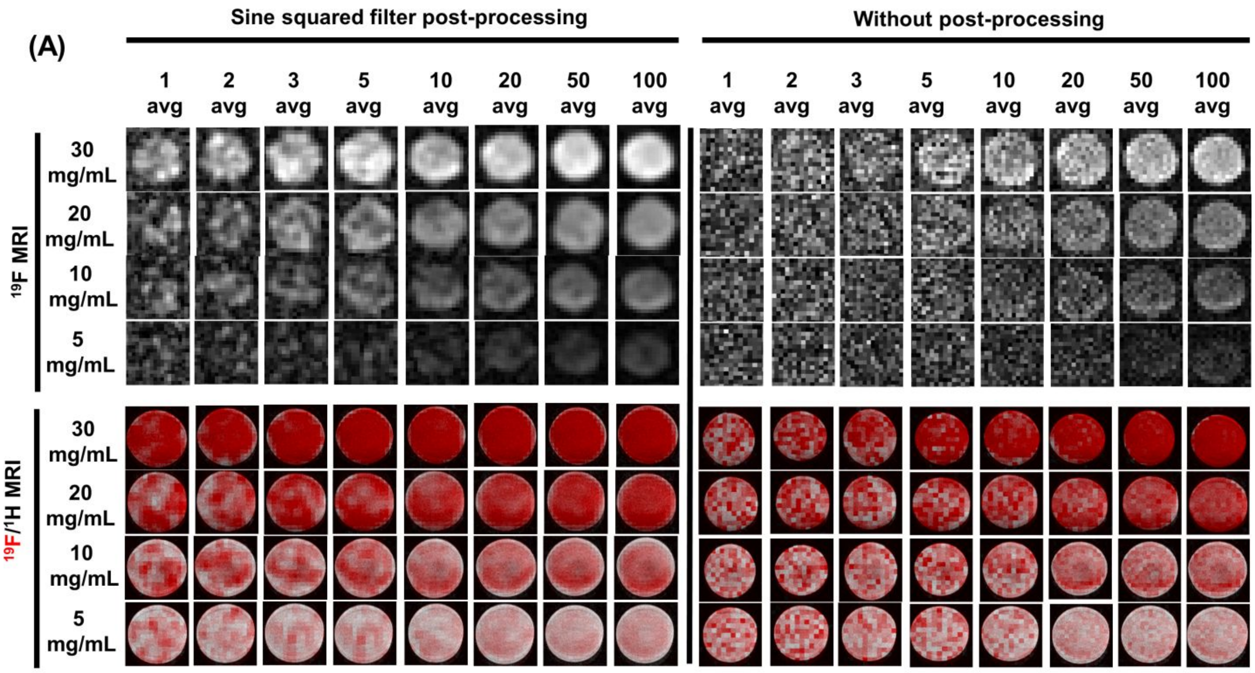

(B)

Acquistion time	No. of averages
4min	1
8min	2
12min	3
21min	5
43min	10
1h 25min	20
3h 30min	50
7h	100

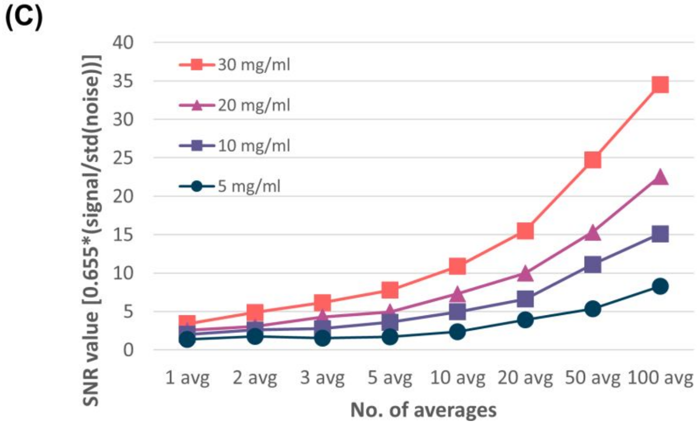

Figure S15. ^{19}F MSME and ^{1}H MRI images (4.7 T) of F5H2 nanoparticles in water at different polymer concentrations and a different number of acquisitions. Overlay images show a ^{19}F MRI signal in red color. (B) Overview of MRI acquisition times. (C) Dependence of ^{19}F MRI SNR on the number of acquisition scans.

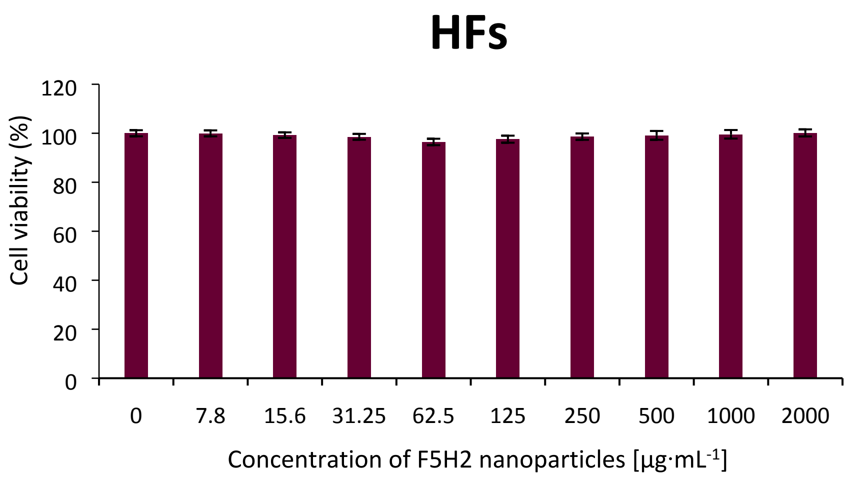

Figure S16. The cytotoxicity evaluation of F5H2 nanoparticles in primary human dermal fibroblasts (HFs) *in vitro*. The cell viability of HFs after 72 hours of treatment with different concentrations of F5H2 nanoparticles was measured with PrestoBlue cell viability reagent. Data are present as means ± S.E.M. of three experiments performed in sextuplicate.

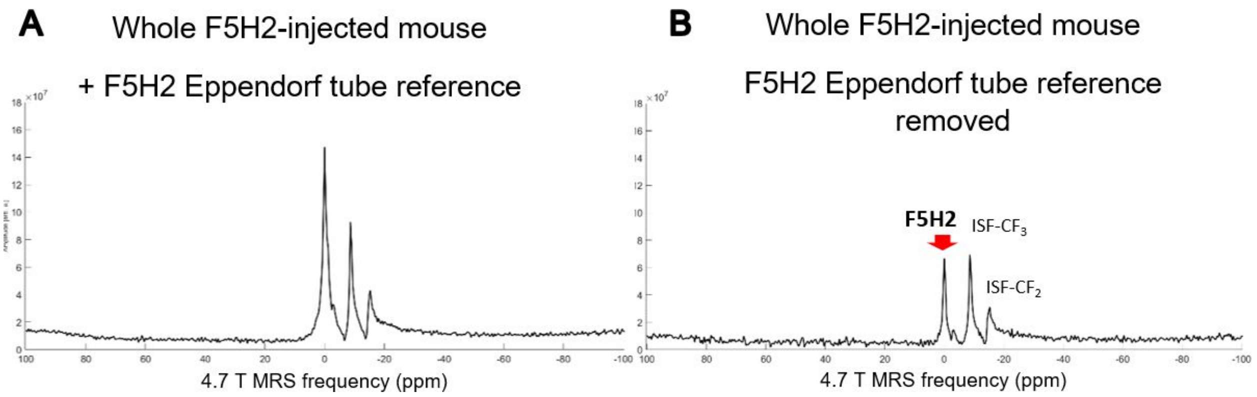

Figure S17. Non-localized *in vivo* ^{19}F MRS measurement of F5H2-injected animal at 4.7 T (A) with, respectively (B) without (right) an F5H2 Eppendorf tube reference used for the MRS peak assignment. Two peaks of isoflurane anesthetic with chemical shift δ = -8.6 ppm (ISF-CF_3) and δ = -15.3 ppm (ISF-CF_2) are clearly separated from the signal originating from the polymer.

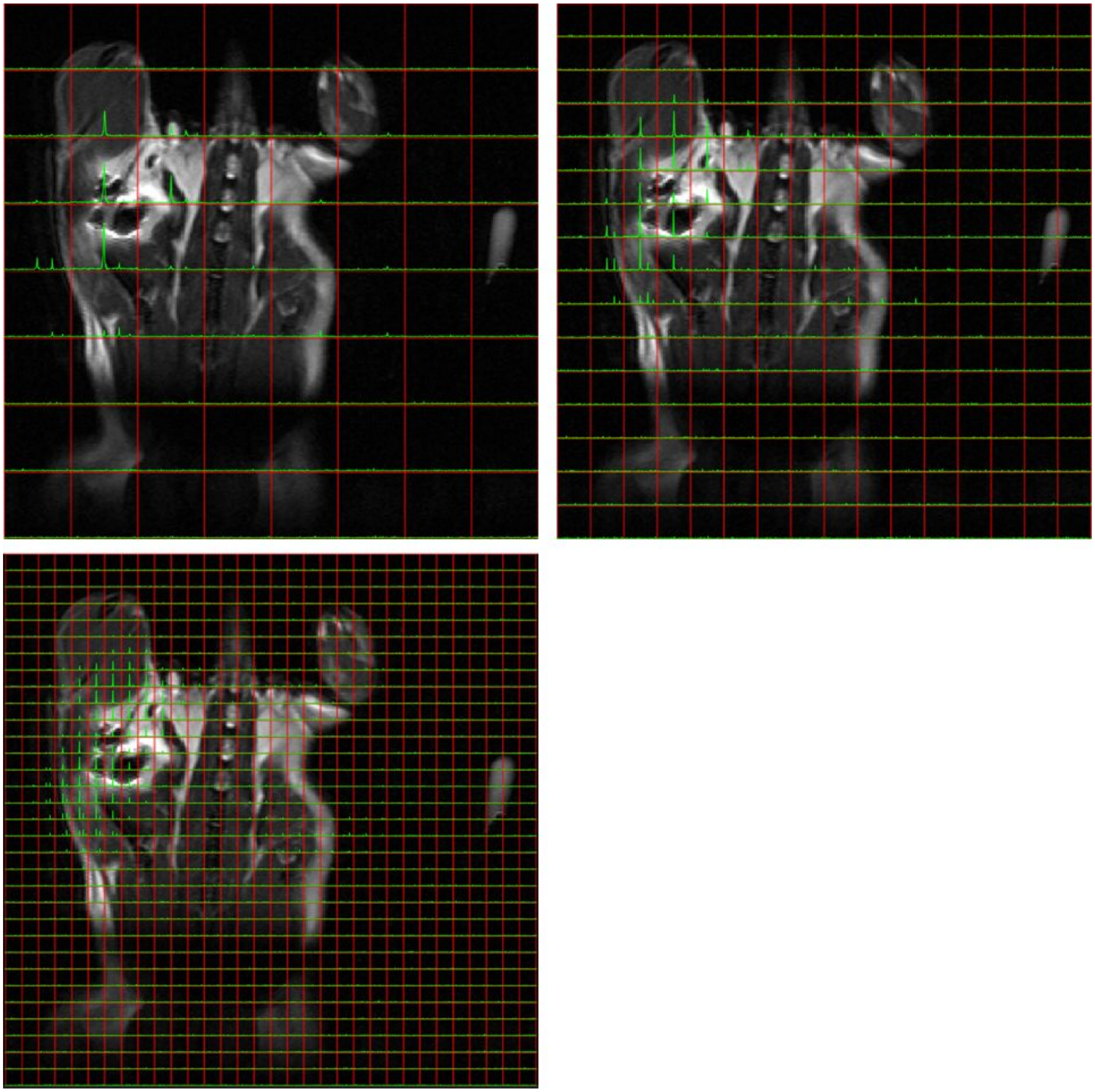

Figure S18. Spatial distribution of F5H2 ^{19}F NMR signal overlaid on ^{1}H MRI (4.7 T) with different grid mesh. Solution of F5H2 (60 mg mL-1, 200 μL) was subcutaneously injected into the right leg of a healthy female Balb/C mouse. The red grid shows CSI voxels and corresponding spectra (green color). The signal intensities from the applied polymer correspond to the CSI image

reconstructed for the polymer frequency range only. No polymer signal was detected in the area corresponding to the left leg.
